# Physicochemical Insight into Coordination Systems Obtained from Copper(II) Bromoacetate and 1,10-Phenanthroline

**DOI:** 10.3390/molecules25225324

**Published:** 2020-11-15

**Authors:** Ewelina Krejner, Tomasz Sierański, Marcin Świątkowski, Marta Bogdan, Rafał Kruszyński

**Affiliations:** Institute of General and Ecological Chemistry, Lodz University of Technology, Zeromskiego 116, 90-924 Lodz, Poland; ewelina.krejner@edu.p.lodz.pl (E.K.); tomasz.sieranski@p.lodz.pl (T.S.); marta.bogdan@dokt.p.lodz.pl (M.B.)

**Keywords:** copper, bromoacetate, coordination compound, UV-Vis, IR, thermal analysis

## Abstract

Two different coordination compounds of copper were synthesized from the same building blocks (1,10-phenanthroline, bromoacetate anions, and copper cations). The synthesis parameters were carefully designed and evaluated to allow the change of the resulting compounds molecular structure, i.e., formation of mononuclear (bromoacetato-*O*,*O*’)(bromoacetato-*O*)aqua(1,10-phenanthroline-*N*,*N*’)copper(II) and dinuclear (μ-bromido-1:2κ^2^)bis(μ-bromoacetato-1κ*O*,2κ*O’*)bis(1,10-phenanthroline-*N*,*N*’)dicopper(II) bromoacetate bromoacetic acid solvate. The crystal, molecular and supramolecular structures of the studied compounds were determined and evaluated in Hirshfeld analysis. The UV-Vis-IR absorption and thermal properties were studied and discussed. For the explicit determination of the influence of compounds structure on radiation absorption in UV-Vis range, density functional theory and time-dependent density functional theory calculations were performed.

## 1. Introduction

The synthesis of coordination compounds with the intended structure is a challenging process [1,2]. Metal centers can adopt diverse coordination numbers (c.n.) and may posses various oxidation states [3,4,5]. As a specific example, a copper cation can create classical coordination compounds with the c.n. from 2 [6] to 8 [7], and with over a dozen of covalently bonded atoms for copper clusters [8]. For c.n. = 6, and for six different ligands, 30 different isomers may be created. When the c.n. is higher, the number of possible isomers increases drastically. The copper usually exists in the form of Cu^+^ and Cu^2+^ cations, but species such as Cu^3+^ and Cu^4+^ also exist [9,10]. Each can adopt a whole variety of coordination polyhedra [11,12,13,14]. Even small changes in the structure of a coordination unit may significantly influence the physicochemical properties of a compound.

The coordination compound formation in a solution depends on many factors such as stoichiometry, concentration, ionic strength, solvent type, and temperature [15,16]. Typically, different coordination species exist in an equilibrium in a solution, but not necessarily the dominant one can crystallize. Hence, a proper crystallization environment may allow the production of forms faintly existing or not detectable in the solution (e.g., due to the ions’ replacement on the crystal surface during crystal formation).

In this work, the formation of different compounds from the same building blocks was studied. As a ligand, the bromoacetate anion was selected. It possesses multifunctional coordination possibilities, and it may be quickly transformed into different species within the specific reaction environment [17]. Bromoacetate anions convert to bromide ions and glycolic acid at elevated temperature or increased concentration/ionic strength. This conversion permits the formation of unique compounds that one cannot synthesize from respective salts [18] and may alter the simple mononuclear coordination moieties to more complex. The additional chelating ligand was used to avoid simple infinite propagation of the coordination via a double bridged carboxylate group. For this purpose, the 1,10-phenanthroline (phen) was selected as its usefulness for breakage (partially or totally) of multinuclear compounds formation was already proven in different systems [19,20,21,22]. The applied strategy allowed the synthesis of two new coordination compounds from building blocks used previously to synthesize mononuclear compounds. This work broadens the knowledge of constructing different compounds from the same building units and shows how the alteration of metal centers coordination influences compound properties changes (electronic and thermal). It also highlights difficulties that one may encounter during the analysis of the spectroscopic properties of open-shell systems.

## 2. Results and Discussion

### 2.1. Synthesis

The reaction between copper(II) bromoacetate and 1,10-phenanthroline (phen) leads to the subsequent formation and crystallization of two new coordination compounds, i.e., (bromoacetato-*O,O*’)(bromoacetato-*O*)aqua(1,10-phenanthroline-*N,N*’)copper(II) (**1**) and (μ-bromido-1:2κ^2^)bis(μ-bromoacetato-1κ*O*,2κ*O’*)bis(1,10-phenanthroline-*N*,*N*’)dicopper(II) bromoacetate bromoacetic acid solvate (**2**), or to the formation of only one compound (**1**), depending on the starting concentration of the solution. The larger concentration leads to partial decomposition of the bromoacetate ions due to the larger ionic strength of a solution and subsequently allows the formation of compound **2** in the synthesis S2. An increase in ionic strength allows the decomposition of bromoacetate ions and speeds up this process [17,18]. Consequently, **1** and **2** were formed simultaneously in solution S2B. The bromide ions were detected in the synthesis S2B after stirring. However, they were absent even at the end of crystallization after the synthesis S1B. The earlier formation of the crystals of **2** than the crystals of **1** originates from different solubility of both compounds in water (approximate values 2.1 and 8.0 g/100 cm^3^ of used solvent mixture, respectively). After forming and separating the crystals of **2**, the bromide ions were absent in the solution (the formed bromide ions were utilized in the formation of **2**, and further decomposition of bromoacetate ions did not occur due to the lowering of solution ionic strength after crystallization of **2**). The phase purity of each crystal fraction was confirmed by comparing the high-resolution IR spectra registered for the crystal with the determined structure and for the bulk sample (the differences allowing unambiguous assignment of phases are described in paragraph 3.4 and collected in Table S4). The studied compounds possess 1:1 M:L stoichiometry, but they were formed only from 2:1 M:L stoichiometry of the substrates. Application during synthesis 1:1 M:L stoichiometry led to the formation of literature-known [CuBr(phen)_2_]^+^•Br^−^ hydrate [23]. This is caused by large stability constants of Cu-phen systems (log*β*_Cu(phen)_ = 9.25, log*β*_Cu(phen)2_ = 16.0, log*β*_Cu(phen)3_ = 21.35) [24], and necessity of ligand deficiency in a reaction system for the formation of the compound with 1:1 M:L stoichiometry, i.e., shifting of equilibria toward the formation of Cu(phen)^2+^ species (instead of Cu(phen)_2_^2+^ and Cu(phen)_3_^2+^ species) by decreasing the concentration of phen. In the literature, the description of the synthesis of [Cu(BrCH_2_COO)_2_(phen)] compound in hydrothermal conditions can be found [25]. Nevertheless, repeating this synthesis (for details see Supplementary Materials) leads to the formation of [CuBr(phen)_2_]^+^•Br^−^ hydrate [23] instead of [Cu(BrCH_2_COO)_2_(phen)]. It might originate from different conditions in the original and repeated syntheses (current work), as in the original paper [25] experimental details (e.g., temperature and time of reaction, a volume of used vessel, reached pressure within the vessel) were not provided.

### 2.2. Structural Analysis

**Figure 1:**
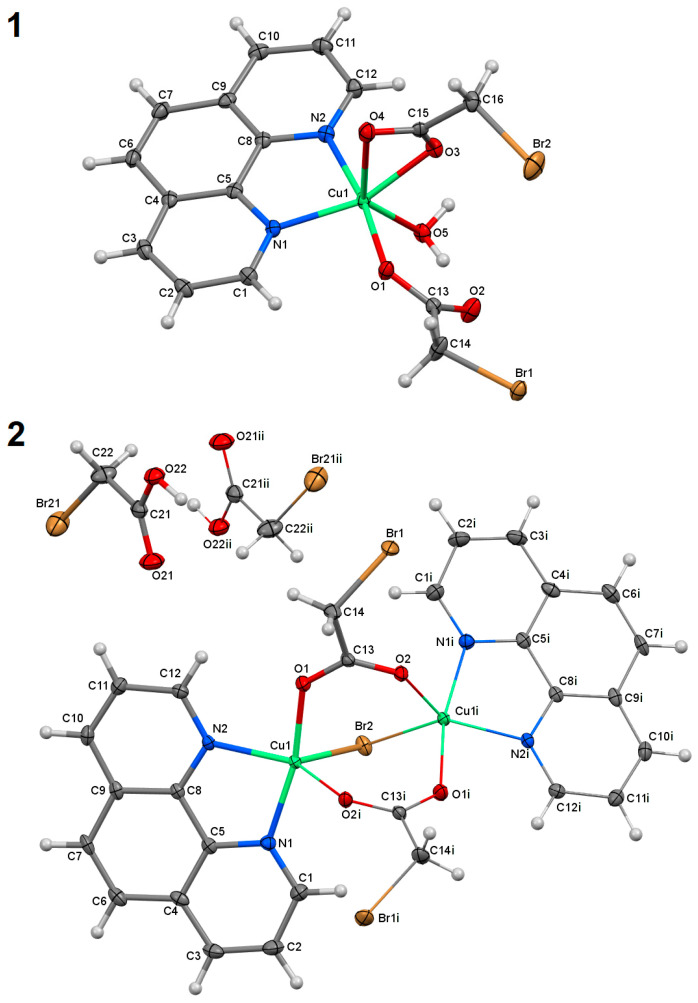
Solid state structures of the studied compounds, with atom numbering scheme, plotted with 50% probability of displacement ellipsoids of non-hydrogen atoms. Hydrogen atoms are plotted as spheres of arbitrary radii. The symmetry generated atoms are indicated by i and ii letters [symmetry code: (i) −x + 1, y, −z + 1.5; (ii) −x + 0.5, −y + 0.5, −z + 1]. The occupancy of hydrogen atom bonded to O22 is set on 0.5 (see Section 3.2).

Compound **1** is a molecular system possessing one coordination moiety located in an asymmetric unit and no ligands in an outer coordination sphere (Figure 1). Compound **2** is distinctly more complex. Its coordination moiety is dinuclear and occupies two asymmetric units (Figure 1). All except one atom of **2** lie in general positions. The presence of the twofold rotation axis (special position e of *C*2/*c* space group with multiplicity 4) going through Br2 ion causes the dinuclear complex cation to be composed of two symmetry dependent bridging bromoacetate ions, two chelating phen molecules, two copper cations and one bridging bromide ion. The observed dinuclear triple-bridged core is unique among copper-phen coordination compounds (among 3415 structurally determined copper-phen compound [26] none possesses bridges formed by two carboxylate ions and one halogen ion). The outer coordination sphere of **2** contains one bromoacetate ion balancing the complex cation charge and one neutral bromoacetic acid molecule (each existing in different asymmetric units). The inversion center (special position d with multiplicity 4) between neighboring bromoacetate anion and bromoacetic acid molecules leads to disorder of the carboxylic hydrogen atom equally over two positions and the formal presence of half of the anion as well as half of the acid in one asymmetric unit.

**Figure 2:**
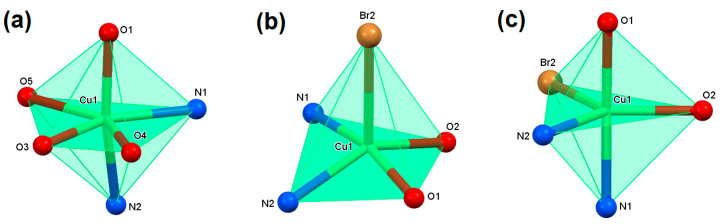
Coordination polyhedra of the compound **1** (**a**) and **2** (**b**–**c**). For a description, see the text.

The copper atom of **1** is six-coordinated and adopts a distorted tetragonal bipyramid geometry with O1 and N2 atoms located at the polyhedron apexes (Figure 2a) [27]. The Cu1-O4 coordination bond (Table 1) is distinctly longer than other ones, but it still falls in the range of the sum of van der Waals radii (equal 2.9 Å according to Bondi or 3.2 according to Zefirov) [28,29]. The bond valence analysis [30,31,32,33] shows that BVS (the sum of bond valences, Table 1) in **1** is equal to 1.894 for presumed c.n. = 5 and 1.974 for c.n. = 6. It proves the conclusion about the six-coordinated copper atom of **1** based on the bond lengths. The central atoms of **2** are five-coordinated and exist in a transitional coordination geometry between square pyramidal (Figure 2b) and trigonal-bipyramidal (Figure 2c) [34]. The observed distortion from an ideal polyhedron geometry observed in both compounds is caused by the rigid and flat structure of the chelating phen molecules and the restraints provided by the chelating (in **1**) and bridging (in **2**) carboxylate anions. The large structural strains occurring around central atoms disallow occupation of ideal positions by the coordinating atoms of the ligands. The chelating bromoacetate anion of **1** asymmetrically bonds to the copper cation, and the bond length difference is 0.77 Å (Table 1). Oppositely, in **2** the bridging bromoacetate anion shows symmetricity of the formed coordination bonds.

The supramolecular structures of the studied compounds are stabilized by O-H•••O, C-H•••O and C-H•••Br hydrogen bonds (Table S1). In **1**, the unitary graph set of classical hydrogen bonds comprises S(6) and R_2_^2^(8) motif. In **2,** it contains only one finite D(2) motif. Both S and R motifs engage water molecules as hydrogen bond donors and carboxylate ions as acceptors. The S motif is formed by O-H•••O interaction between water molecules and carboxylate ions coordinated to the same copper cation while R motif is formed by O-H•••O hydrogen bonds between the neighboring coordination units, linking them into a supramolecular dimer. In **2**, the present D(2) motif is formed only by the interaction between species present in the outer coordination sphere—a characteristic anti–anti type arrangement for carboxylate•••carboxylic acid interactions [35]. The molecules of **1** are stacked to the piles extending along [100] crystallographic axis via π•••π interactions between aromatic rings of phen (Table S2, Figure S6). Each six-membered ring interacts with a total of four neighboring rings. One of these rings belongs to one of the adjacent phen molecules in the pyridine case, and three others belong to second nearby phen molecules. Each benzene ring always interacts with two rings of each neighboring phen molecule. The molecules of **2** are stacked to the layers expanding along (1 0 0) crystallographic plane due to the presence of two phen ligands in the coordination unit (the phen molecules themselves are assembled to piles propagating along [010] crystallographic axis via π•••π interactions between aromatic rings of phen; Figure S6). In **2,** only one six-membered ring (i.e., the pyridine ring containing N1 atom) interacts with four neighboring rings, and two other rings interact with six adjacent rings of two symmetry-related phen molecules. In both compounds, the subsequent phen molecules are packed alternatively in the crystal net (succeeding phen molecules are reversed along bond linking pyridine rings). The intermolecular interactions were also analysed based on Hirshfeld surfaces and fingerprints (see Supplementary Materials for details, Figures S1–S5 and S7–S8).

### 2.3. UV-Vis Spectra Analysis

**Figure 3:**
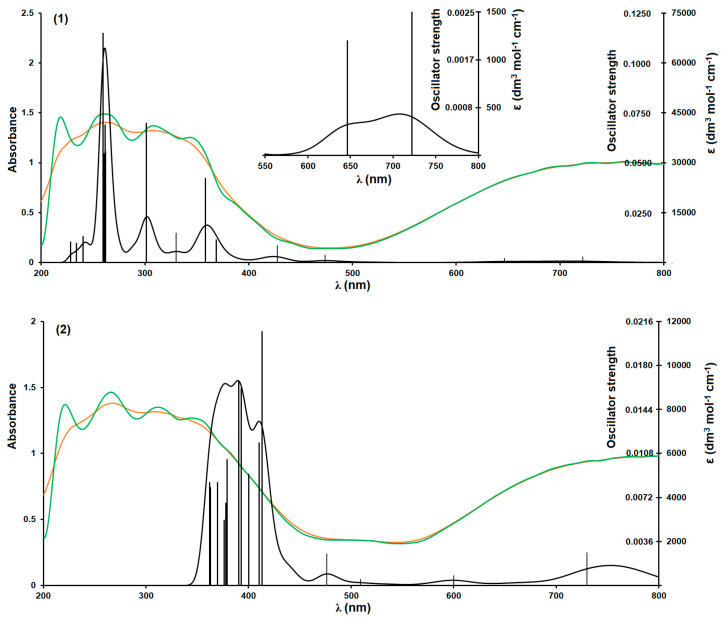
Experimental (orange-registered, green-deconvoluted) and calculated (black) UV-Vis spectra of the studied compounds (**1** and **2**). The most important oscillator strengths are shown as vertical black lines.

The calculated spectrum of **1** is similar to the experimental one. Noticeable differences exist in the case of **2** (Figure 3, Figure S14, Table 2). TD-DFT works better for closed-shell systems [36]. Both **1** and **2** contain open-shell centers. However, the case of **2** is more complicated. The centers in **2** can produce a singlet-state molecule with unpaired electrons of two Cu^2+^ possessing opposite spins, or they can form a triplet state molecule with unpaired electrons of two Cu^2+^ possessing the same spins. Therefore, the spectrum of **2** was more challenging to reproduce. All applied approaches (Figure S14) led to different spectra. For UB3LYP approach, the number of the observed maxima and their mutual arrangement are accurately reproduced and reasonably reflect the experimental ones. Yet, this approach alone does not give a system for which its electronic state’s energy is the lowest. Only the optimization of a wave function led to a stable wave function and gave a state with the lowest energy (an antiferromagnetic singlet state, Table S3).

**Figure 4:**
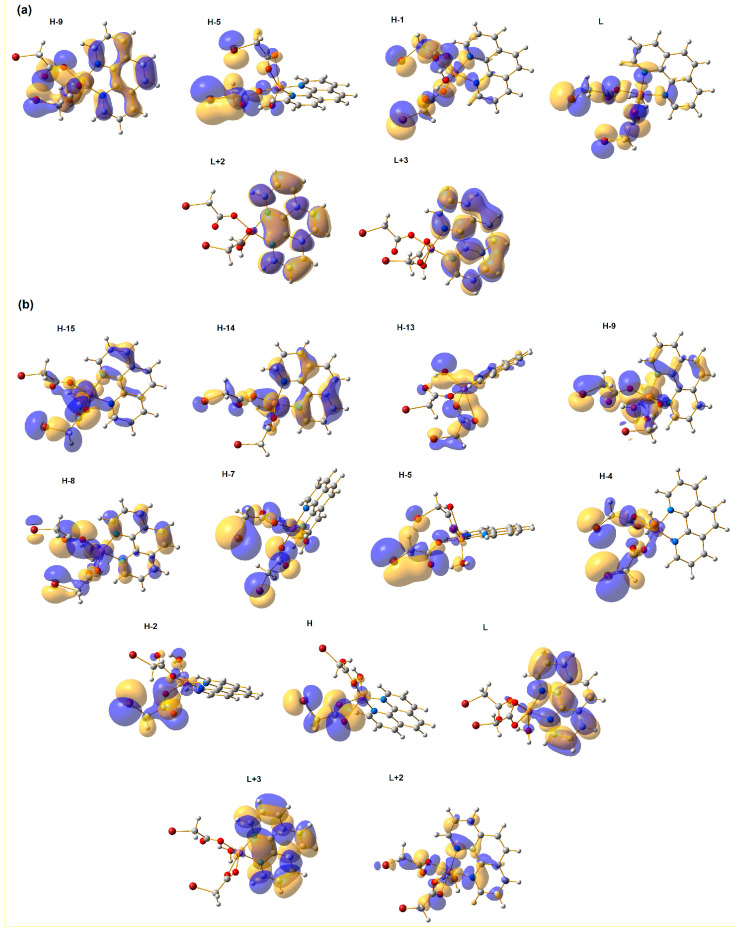
Calculated molecular orbitals of compound **1**: α (**a**) and ß (**b**). H letter indicates HOMO, L-LUMO, and ±(number) represents subsequent orbitals above HOMO and LUMO, respectively.

**Figure 5:**
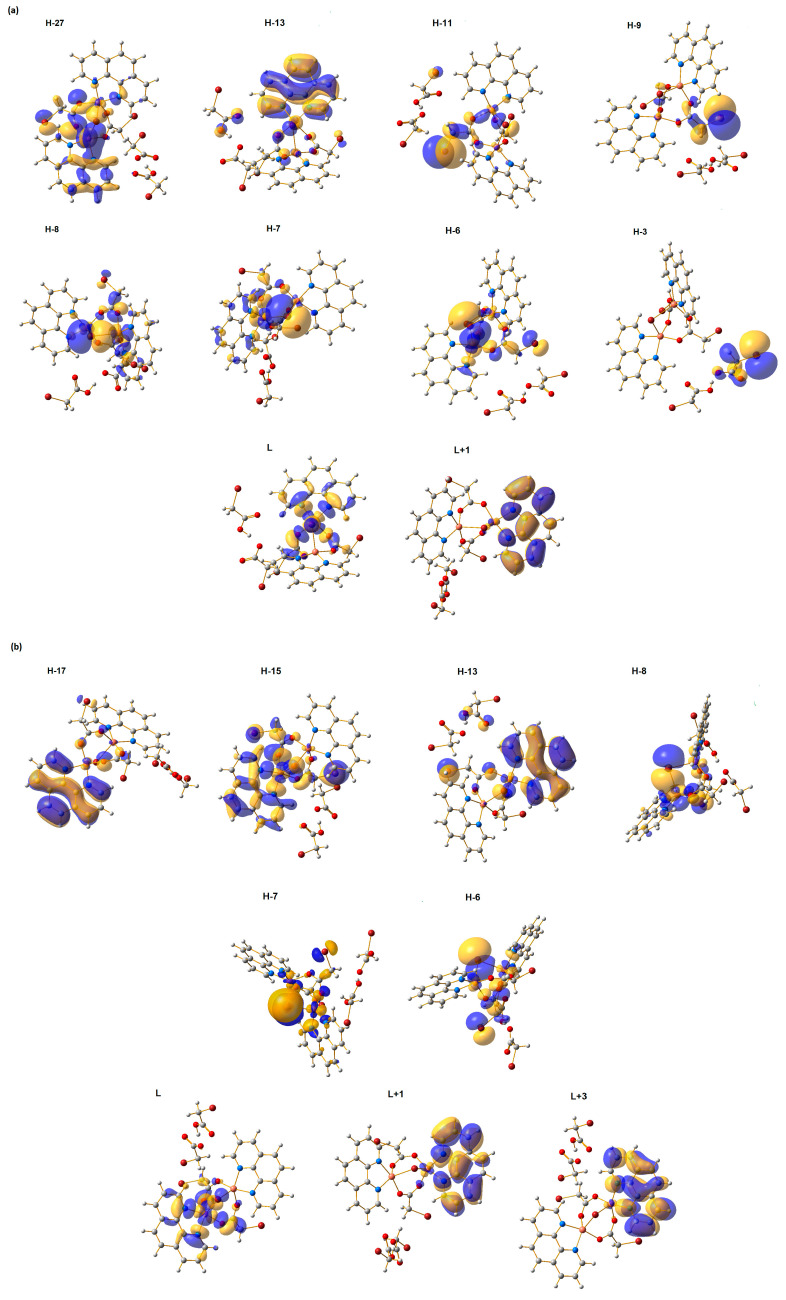
Calculated molecular orbitals of compound **2**: α (**a**) and ß (**b**). H letter indicates HOMO, L-LUMO, and ±(number) represents subsequent orbitals above HOMO and LUMO, respectively.

Three first absorption maxima of the calculated spectrum of **2** are shifted towards larger wavelengths (of around 100 nm) comparing to experimental ones. The broad absorption maximum observed above 500 nm is well reproduced both for **1** and **2** (Figure 3, Table 2). Since the studied compounds were treated with UB3LYP, two separate sets of orbitals (α and β, Figure 4 and Figure 5) were generated. For both compounds, the electronic transitions involve complex molecular orbitals. Generally, the first two maxima are mostly n→π* transitions related to ligand-to-ligand charge transfer (LLCT). They involve the non-bonding orbitals of bromoacetate and bromide anions and antibonding π* orbitals of phen ligands (Table 2). In a solid-state spectrum of pure phen, those maxima are observed at slightly lower wavelengths, and they are associated with π→π* transitions [18,21]. The next absorption maximum is mostly caused by ligand-to-metal charge transfer (LMCT) transitions (Table 2, Figure 4 and Figure 5). The contribution of LMCT seems to be larger in **2** (Table 2)–the respective LUMO orbitals involves mostly electrons localized near the metal centers (Figure 5). The corresponding maximum in a solid-state spectrum of pure phen is observed for slightly lower wavelength numbers (Table 2). Similarly to the previous maxima, it is associated with π→π* transitions. The next maximum is related to LLCT and is seen as *n*→π* transitions involving lone pair orbitals of bromoacetate and bromide anions, lone pair orbitals of water molecules, and π antibonding orbitals of phen (Table 2). For the solid-state spectrum of phen that maximum corresponds to two maxima (Table 2) associated with *n*→π* transitions. As other phen maxima, they are observed at smaller wavelengths than the studied compounds’ maxima. The last maxima are produced by multiple complex orbital transitions involving d orbitals of copper cations, non-bonding orbitals of bromoacetic acid/bromoacetate ions, antibonding σ orbitals of phen ligands and antibonding σ orbitals of bromoacetic acid/bromoacetate ions (Table 2, Figure 4 and Figure 5).

The studied compounds contain a ligand (phen) possessing fluorescence properties in a free state. The number of emitted photons by phen may increase due to changes in the orbitals energy upon coordination. However, the bromine’s introduction into the molecular systems diminishes or quenches the fluorescence [37,38]. Both **1** and **2** show no significant fluorescence, i.e., only a few photons were registered during measurement, and they can be considered instrument artifacts coming from second-order diffraction by grating monochromators (some intensity appears in the area of λ_em_ = 2 λ_ex,_ Figure S9). Thus, it can be stated that the presence of bromoacetate ions in the studied compounds is a sufficient condition for the total quenching of phen fluorescence.

### 2.4. IR Spectra Analysis

The ATR-IR spectra of the studied compounds contain sets of bands characteristic for bromoacetate anions [39] and phen molecules [40,41] (Table S4, Figure S10). The most important bands of carboxylate anions correspond to stretching vibrations of carboxylate groups. A binding mode of COO^-^ can be estimated based on the separation parameter Δ*ν*, which is a difference between the wavenumbers of *ν_as_* COO and *ν_s_* COO [42,43,44]. In both studied compounds, the bromoacetate anions exhibit two different binding modes, i.e., asymmetric bidentate chelating and monodentate in **1**, and bidentate syn-syn bridging and non-coordinating in **2**. Rarely, in similar cases, it is possible to distinguish two bands of *ν_as_*, which allow calculation of Δ*ν* independently for both binding modes of COO^-^ [45]. For the studied compounds, only one *ν_as_* band is present, and additionally, it is concealed by the bands of the stretching CC and CN vibrations of phen (existing in the same region). In the case of **1**, the chelating anions exhibit strong asymmetricity of O-Cu bond lengths, causing one of these bonds to be significantly stronger than the second. As a result, such chelating mode becomes similar to the monodentate mode, and thus only one ν_as_ band exists. In the structure of **2**, there are two bridging bromoacetate anions per one non-coordinating anion. Thus the *ν_as_* band corresponds very likely to the first ones. The Δ*ν* values equal to 182 and 204 cm^−1^, respectively for **1** and **2**, agree with literature data for the particular binding mode of bromoacetate, i.e., monodentate: 183 cm^−1^ [46]^,^ 191 cm^−1^ [47] and bidentate syn-syn bridging: 201 cm^−1^ [48]. The spectrum of **2** confirms the presence of bromoacetic acid in the compound structure. The most evident is the band at 1738 cm^−1^ produced by the stretching C=O vibrations of the carboxylic group [49] (Table S4). Most phen bands are shifted toward larger wavenumbers (compared to pure phen) due to bonds stiffening caused by the formation of a five-membered chelating ring with copper cation. The phen coordination also leads to the differentiation of energy of the same types of oscillators resulting in the splitting of corresponding bands of pure phen, e.g., *ν* CC, *ν* CN, *δ-α* CH at 1345 cm^−1^ or *δ-γ* CH at 1092 cm^−1^ (Table S4).

### 2.5. Thermal Analysis

**Scheme 1:**
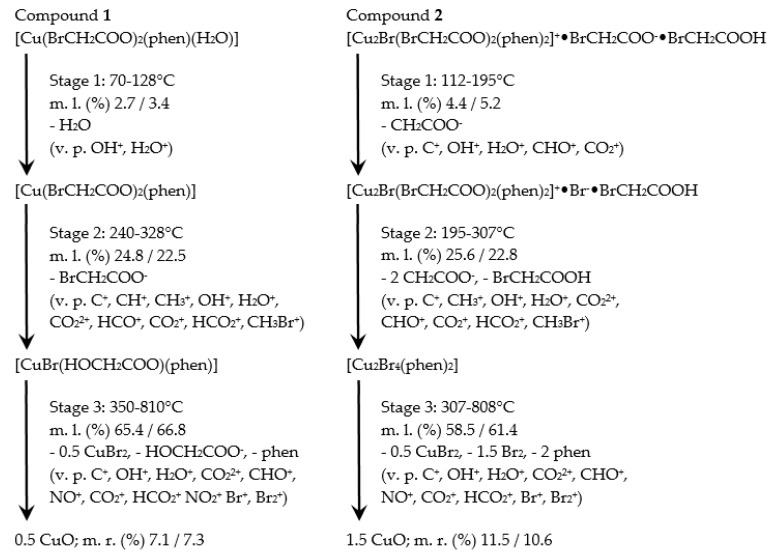
Thermal decomposition stages of the studied compounds (v. p. = volatile products established from mass spectra; m. l.—experimental/theoretical mass loss; m. r.—experimental/theoretical mass residue).

Compound **1** decomposes in three, well separated, step-processes (Scheme 1, Figure S11). The first stage is dehydration (Figure S12). The dehydrated compound is stable from 128 °C to 240 °C. In this temperature range, the exothermic process occurs without a mass loss (at 145 °C, Figure S11). It is a transition resulting from structure rearrangement after water evacuation. Next, one of the bromoacetate anion decomposes, and process products are removed. Simultaneously, the second carboxylate undergoes dehalogenation with the formation of glycolate anion and bromide [18]. The last stage comprises the oxidation of glycolate and phen, together with the removal of half of the copper content in the form of copper dibromide, which sublimates at high temperatures [50]. The sublimated CuBr_2_ decomposes in a mass spectrometer, which is visible in the mass spectrum as characteristic for bromine, *m*/*z* signals equal to 80 and 160 (Figure S12). The final product is pure copper oxide. The decomposition of **2** begins with the degradation of the non-coordinating bromoacetate, which is less stable than the bromoacetic acid [18,51]. After that stage, the acid’s oxidation and the rest of the anions occurs. The phen molecules decompose in the last stage. Similarly to the decomposition of **1**, the part of the copper content is also removed (Figure S13) and the final product is copper oxide.

## 3. Materials and Methods

### 3.1. Synthesis

All reagents were bought from Sigma-Aldrich (St. Louis, MO, USA) and were analytical grade. The analytical grade water was prepared from improved drinking water via combined reverse osmosis and ion exchange processes.

#### 3.1.1. Preparation of Copper(II) Bromoacetate Solutions

Copper(II) bromoacetate solutions was prepared (procedure S1A) by suspending the dicopper carbonate dihydroxide (0.25 mmol, 0.0553 g) in a water solution of bromoacetic acid (0.60 mmol, 0.0834 g of acid dissolved in 5.00 cm^3^ of water). The mixture was stirred at room temperature (to avoid decomposition of bromoacetate ions) for 30 min (rotation speed was 300 rpm). Next, it was filtered (to remove an unreacted excess of dicopper carbonate dihydroxide), and the solid residue was washed with 1.25 cm^3^ of water. The combined solutions were used in subsequent synthesis. The synthesis was repeated at a larger scale (procedure S2A) with the following alterations of compound/solvent amounts: dicopper carbonate dihydroxide, 2.5 mmol (0.5528 g); bromoacetic acid, 6.0 mmol (0.8337 g) dissolved in 25.0 cm^3^ of water; water used for washing, 5.0 cm^3^.

#### 3.1.2. Synthesis of Coordination Compounds

The coordination compounds were synthesized in a reaction between the copper(II) bromoacetate and 1,10-phenanthroline (phen). The 2:1 molar ratio of metal to ligand (M:L) was applied. The above described (procedure S1A) water solution of copper bromoacetate (0.3 mmol, 6.25 cm^3^) was mixed with the solution of phen (0.15 mmol, 0.0297 g dissolved in 5.0 cm^3^ equivolume mixture of methanol and water; procedure S1B). The resulting solution was stirred vigorously (rotation speed was 1500 rpm) at room temperature for 30 min and it was left to crystallize at 20 °C. Light blue prism crystals of [Cu(BrCH_2_COO)_2_(phen)(H_2_O)] (**1**) grew after one week (mass: 0.0742 g, yield_(phen)_ = 92%). The synthesis was repeated at a larger scale (procedure S2B) with the following alterations: the water solution of copper bromoacetate (3.0 mmol, 30 cm^3^) from procedure S2A was used, and the solution of phen contained 1.5 mmol, 0.2973 g of the substance dissolved in 10.0 cm^3^ of solvent mixture. Deep blue plate crystals of [Cu_2_Br(BrCH_2_COO)_2_(phen)_2_]^+^ BrCH_2_COO^−^ BrCH_2_COOH (**2**) formed after 3 days (mass: 0.4117 g, yield_(phen)_ = 49%) were separated by filtration and after one week the light blue prism crystals of **1** were formed (mass: 0.3387 g, yield_(phen)_ = 42%). Elemental analyses included C, H, N, O elements were carried out using Vario EL III CHNOS Elemental Analyzer (Elementar, Langenselbold, Germany). Results for **1** [calculated/found (%)]: C 35.74/35.36; H 2.62/2.31; N 5.21/5.09; O 14.88/15.02 and for **2**: C 34.31/34.01; H 2.25/2.11; 5.00/4.88; O 11.43/11.54.

### 3.2. Crystal Structure Determination

X-ray intensity data of the studied compounds were collected at 100.0(1) K, on a Rigaku Synergy Dualflex automatic diffractometer (Rigaku Corporation, Tokyo, Japan) equipped with Pilatus 300K detector and micro-focus sealed PhotonJet X-ray tubes generated monochromated Mo*K_α_* (λ = 0.71073 Å) or Cu*K_α_* (λ = 1.54184 Å) radiation, with shutterless ω scan mode (the optimal radiation wavelength was selected according to preliminary measurement results performed for both wavelengths: Cu*K_α_* for **1** and Mo*K_α_* for **2**). Lorentz, polarization, and empirical absorption correction (using spherical harmonics, implemented in SCALE3 ABSPACK scaling algorithm) corrections were applied during the data reduction. The structure was solved by dual-space algorithm. All non-hydrogen atoms were refined anisotropically using full-matrix, least-squares technique on *F*^2^. All hydrogen atoms were found from difference Fourier synthesis after ten cycles of anisotropic refinement. Carbon bonded hydrogen atoms were refined as “riding” on the adjacent atom with geometric idealization after each cycle of refinement. Individual isotropic displacement factors of carbon bonded H atoms were set to be equal to 1.2 times the value of equivalent displacement factors of the parent atoms. Oxygen bonded hydrogen atoms positions were freely refined, and their individual isotropic displacement factors were fixed to 1.5 of the value of equivalent displacement factors of the parent atoms. For compound **2**, the occupancy factor of the carboxylic group hydrogen atom (H22O) was set to 0.50 as the O22–H22O group interacts with the symmetry generated O22(−x + 0.5, −y + 0.5, −z + 1) atom and thus, the H atom can exist only at one of these O22 atoms. Thus, the H atom is equally disordered by symmetry over two positions and exists either in the asymmetric unit or as the symmetry generated H atom. Existence in both positions (i.e., at both neighboring molecules) is impossible due to the formation of an unacceptably short H22O•••H22O(−x + 0.5, −y + 0.5, −z + 1) distance. The SHELXT [52], SHELXL [53] and SHELXTL [54] programs were used for all calculations. Atomic scattering factors were taken from International Tables for Crystallography [55]. Details concerning crystal data and refinement are given in Table 3.

CCDC 1990904 (compound **1**) and 1990905 (compound **2**) contain the supplementary crystallographic data for this paper. These data can be obtained free of charge via http://www.ccdc.cam.ac.uk/conts/retrieving.html (or from the CCDC, 12 Union Road, Cambridge CB2 1EZ, UK; Fax: +44-1223-336033; E-mail: deposit@ccdc.cam.ac.uk)

### 3.3. Other Physical Measurements

The UV-Vis diffuse reflectance spectra were recorded on a Jasco V-660 spectrometer (Jasco, Easton, MD, USA), in the spectral range 200–850 nm, using spectralon [56] as a standard with 100% reflectance. The three-dimensional fluorescence spectra were recorded on a Jasco FP-6300 spectrofluorometer (Jasco, Easton, MD, USA), with solid samples directed at an angle of 30° to the incident beam. The excitation and emission wavelength ranges were 220–640 nm and 230–740 nm, respectively. The data pitch and bandwidth were 1 nm on both monochromators. The ATR-IR spectra of the coordination compounds were recorded on a Bruker INVENIO-R spectrometer (Bruker Optik GmbH, Ettlingen, Germany) in the spectral range 4000–400 cm^−1^. The thermal analyses were carried out with a Netzsch STA 449 F1 Jupiter thermoanalyzer (Netzsch-Geratebau GmbH, Selb, Germany) coupled with a Netzsch Aeolos Quadro QMS 403 mass spectrometer (Netzsch-Geratebau GmbH, Selb, Germany). Samples were heated in corundum crucibles up to 1000 °C, with a heating rate 10 °C∙min^−1^ in synthetic air (20% O_2_, 80% N_2_) flow.

### 3.4. Quantum-Mechanical Calculations

The excited states of the studied coordination compounds have been calculated for X-ray determined coordinates using TD-DFT method. Input structural models were prepared with Mercury CSD 4.3.0 [57] computer program (Cambridge Crystallography Data Centre, Cambridge, UK). Positions of hydrogen atoms have been normalized by moving them along the covalent bond vector (X→H) to the X-H distance equal to the average neutron diffraction value. In each case, as an input, a coordination unit of a respective compound was used. For **2**, the species occupying the outer coordination sphere were included. All calculations were performed utilizing Gaussian09 rev. D.01 (Gaussian Inc., Wallingford, CT, USA) [58] with B3LYP functional and employing 6–31++g(2d,2p) basis set of Pople et al. [36,59]. The absorption maxima of **2** were not accurately reproduced treated the whole system as a singlet or triplet state. Hence, it was calculated using unrestricted DFT (UB3LYP) to model the antiferromagnetic coupling of two Cu(II) doublet state centers (with α and β spins respectively so that all the α electrons could cancel all of the β ones). To ensure that the open singlet state system was the one with the lowest energy, the calculation was repeated using the wave function’s optimization. This process led to a stable wave function and further decreased the system energy (Table S3). The resulted electronic state was then used in TD-DFT calculation. The number of calculated transitions was set to 130 to cover all the experimentally founded bands. The calculated excited states’ assignment to the observed experimental maxima was based on the comparison of excitation energies and the oscillator strengths/intensities of the corresponding maxima. The analysis of the character of respective orbital excitations was based on orbital contour plots. Hirshfeld surface maps and the studied compounds’ finger plots were generated using Crystal Explorer 17.5 [60].

## 4. Conclusions

Two structurally different coordination compounds were synthesized in the reaction between copper bromoacetate and phen used in 2:1 molar ratio. The formation of these compounds is dependent on substrates concentrations (ion strength of solution). Compound **2** is possible to obtain only above a specific concentration, for which ion strength is large enough to decompose bromoacetates and release bromides to the solution. The structural diversity of the studied compounds causes dissimilarities of spectral and thermal properties. This work also highlights the difficulties that may be encountered while analyzing the spectroscopic properties of open-shell systems. Dealing with a system in which one would assume some open shell character checking wave function stability should always be considered. Treatment of the system as open-shell, without checking the wave function stability, does not always allow to obtain a system with the lowest energy.

## Figures and Tables

**Table 1:** Selected structural data of the studied compounds.

i—j	d_ij_ (Å)	*ν*_ij_ (v.u.)	i—j—k	α_ijk_ (°)	i—j—k	α_ijk_ (°)
compound **1**						
Cu1—N1	2.0255(17)	0.432 */0.421 ^†^	N1—Cu1—N2	81.77(7)	N2—Cu1—O5	90.27(6)
Cu1—N2	2.0112(17)	0.449 */0.437 ^†^	N1—Cu1—O1	89.34(6)	O1—Cu1—O3	91.68(6)
Cu1—O1	1.9428(14)	0.461 */0.456 ^†^	N1—Cu1—O3	150.23(6)	O1—Cu1—O4	83.82(6)
Cu1—O3	2.0263(14)	0.368 */0.364 ^†^	N1—Cu1—O4	98.38(6)	O1—Cu1—O5	93.17(6)
Cu1—O4	2.7977(14)	0.046 *	N1—Cu1—O5	118.85(6)	O3—Cu1—O4	52.25(6)
Cu1—O5	2.2175(14)	0.219 */0.217 ^†^	N2—Cu1—O1	171.06(7)	O3—Cu1—O5	90.81(6)
			N2—Cu1—O3	96.52(6)	O4—Cu1—O5	142.65(7)
			N2—Cu1—O4	98.40(6)		
compound **2**						
Cu1—N1	2.001(2)	0.449	N1—Cu1—N2	81.81(9)	N2—Cu1—O2 ^i^	139.74(8)
Cu1—N2	2.033(2)	0.412	N1—Cu1—O1	166.12(8)	N2—Cu1—Br2	114.14(6)
Cu1—O1	1.9378(19)	0.462	N1—Cu1—O2 ^i^	88.52(8)	O1—Cu1—O2 ^i^	93.35(8)
Cu1—O2 ^i^	1.9964(18)	0.394	N1—Cu1—Br2	95.42(6)	O1—Cu1—Br2	97.31(6)
Cu1—Br2	2.6198(4)	0.270	N2—Cu1—O1	87.95(8)	O2 ^i^—Cu1—Br2	105.60(5)

Bond valences (*ν*_ij_) calculated with use of parameters for * six-coordinated and † five-coordinated copper(II). Symmetry transformations used to generate equivalent atoms: (i) −x + 1, y, −z + 1.5.

**Table 2:** The most important electronic transitions. H letter indicate HOMO, L-LUMO, α-α orbitals, β-β orbitals, and ±(number) represents subsequent orbitals above HOMO and LUMO, respectively.

Theoretical λ (nm)		E (eV)	*f*	The Most Important Orbitals Involved in Electronic Transitions	Character of Transition	Experimental λ (nm) (Solid State)		
1	2					1	2	phen
228.31		5.4305	0.0104	αH-5→αL+3 βH-15→βL	d(Cu)/n(BrAcO)/σ(BrAcO)→π*(phen) d(Cu)/n(BrAcO)/σ(BrAcO)/π(phen)→d(Cu)/π*(phen)	218.99	220.84	214
233.98		5.2989	0.0096	αH-9→αL+2	d(Cu)/n(BrAcO)/π(phen)→π*(phen)			
240.36		5.1583	0.0130	βH-8→βL+3	d(Cu)/n(BrAcO)/π(phen)→π*(phen)			
	362.12	3.4238	0.0085	βH-17→βL βH-8→βL+3	π(phen)→ d(Cu)/σ*(phen)/σ*(BrAcO) d(Cu)/n(Br)/n(BrAcO)→π*(phen)			
	363.01	3.4154	0.0081	βH-17→βL βH-8→βL+3	π(phen)→ d(Cu)/σ*(phen)/σ*(BrAcO) d(Cu)/n(Br)/n(BrAcO)→π*(phen)			
	369.66	3.3540	0.0085	βH-13→βL	d(Cu)/n(Br)/n(BrAcO)/π(phen)→d(Cu)/σ*(phen)/σ*(BrAcO)			
259.56		4.7767	0.1149	βH-13→βL	d(Cu)/n(BrAcO)→d(Cu)/π*(phen)	261.35	265.62	253
260.46		4.7602	0.0545	αH-1→αL+3	d(Cu)/n(BrAcO)/σ(BrAcO)→π*(phen)			
261.78		4.7362	0.0689	βH-5→βL+3	d(Cu)/n(BrAcO)/σ(BrAcO)→π*(phen)			
262.05		4.7314	0.0555	βH-5→βL+3	d(Cu)/n(BrAcO)/σ(BrAcO)→π*(phen)			
262.65		4.7206	0.0572	αH-5→αL+2	d(Cu)/n(BrAcO)/σ(BrAcO)→π*(phen)			
	376.25	3.2953	0.0054	βH-6→βL+3	d(Cu)/n(Br)/n(BrAcO)→π*(phen)			
	378.12	3.2789	0.0068	αH-13→αL	d(Cu)/n(Br)/n(BrAcO)/π(phen)→d(Cu)/σ*(phen)/σ*(BrAcO)			
	379.21	3.2696	0.0104	βH-6→βL+3	d(Cu)/n(Br)/n(BrAcO)→π*(phen)			
301.36		4.1142	0.0697	βH-9→βL+2	d(Cu)/n(H_2_O)n(BrAcO)σ(BrAcO)/π(phen)→d(Cu)/σ*(phen)/σ*(BrAcO)	308.46	310.77	294
330.05		3.7565	0.0147	βH-7→βL+2 βH-8→βL	d(Cu)/n(H_2_O)n(BrAcO)→d(Cu)/σ*(phen)/σ*(BrAcO) d(Cu)/n(BrAcO)/π(phen)→d(Cu)/π*(phen)			
	390.59	3.1743	0.0169	αH-11→αL βH-15→βL	n(Br)/n(BrAcO)/σ(BrAcO)→d(Cu)/σ*(phen)/σ*(BrAcO) d(Cu)/(Br)/n(BrAcO)/σ(phen)→d(Cu)/σ*(phen)/σ*(BrAcO)			
	392.79	3.1565	0.0163	αH-11→αL	n(Br)/n(BrAcO)/σ(BrAcO)→d(Cu)/σ*(phen)/σ*(BrAcO)			
	400.16	3.0984	0.0092	αH-9→αL	n(Br)/n(BrAcO)/σ(BrAcO)→d(Cu)/σ*(phen)/σ*(BrAcO)			
358.41		3.4593	0.0421	βH-4→βL+2	d(Cu)/n(H_2_O)/n(BrAcO)/σ(BrAcO)→d(Cu)/σ*(phen)/σ*(BrAcO)	344.61 384.41	344.77	324 335
368.93		3.3606	0.0114	βH-5→βL βH-4→βL	d(Cu)/n(BrAcO)/σ(BrAcO)→d(Cu)/π*(phen) d(Cu)/n(H_2_O)/n(BrAcO)/σ(BrAcO)→d(Cu)/π*(phen)			
	410.44	3.0207	0.0118	βH-7→βL+1 βH-8→βL+1	d(Cu)/n(Br)/n(BrAcO)→π*(phen) d(Cu)/n(Br)/n(BrAcO)→π*(phen)			
	413.33	2.9997	0.0210	αH-8→αL+1 βH-7→βL+1	d(Cu)/n(Br)/n(BrAcO)→π*(phen) d(Cu)/n(Br)/n(BrAcO)→π*(phen)			
427.35			0.0085	βH-2→βL+2	d(Cu)/n(H_2_O)n(BrAcO)σ(BrAcO)→d(Cu)/σ*(phen)/σ*(BrAcO)	439.55		
	476.36	2.6027	0.0026	αH-6→αL αH-7→αL	d(Cu)/n(Br)/n(BrAcO)→d(Cu)/σ*(phen)/σ*(BrAcO) d(Cu)/n(Br)/n(BrAcO)→d(Cu)/σ*(phen)/σ*(BrAcO)		516.59	
473.81		2.6167	0.0037	αH-1→αL	d(Cu)/n(BrAcO)/σ(BrAcO)→d(Cu)/n(BrAcO)/σ*(BrAcO)/σ*(phen)	726.94 759.80	728.98 769.21	
646.65		1.9173	0.0020	βH-14→βL+2 βH-13→βL+2	d(Cu)/n(H_2_O)/σ(BrAcO)/π(phen)→d(Cu)/σ*(phen)/σ*(BrAcO) d(Cu)/n(BrAcO)→d(Cu)/σ*(phen)/σ*(BrAcO)			
712.92		1.7391	0.0028	βH-13→βL+2	d(Cu)/n(BrAcO)→d(Cu)/σ*(phen)/σ*(BrAcO)			
949.37		1.3060	0.0028	βH→βL+2	d(Cu)/n(BrAcO)→d(Cu)/σ*(phen)/σ*(BrAcO)			
	509.01	2.4358	0.0005	βH-7→βL	d(Cu)/n(Br)/n(BrAcO)→d(Cu)/σ*(BrAcO)/σ*(phen)			
	599.93	2.0666	0.0008	αH-3→αL	n(BrAcO)/σ(BrAcO)→d(Cu)/σ*(BrAcO)/σ*(phen)			
	730.15	0.0027	0.0026	αH-27→αL	d(Cu)/n(BrAcO)/n(Br)/σ(phen)→d(Cu)/σ*(BrAcO)/σ*(phen)			

Used abbreviations: d(Cu)–d orbital of copper cation, n(BrAcO)–non-bonding orbital of bromoacetate anion, σ(BrAcO)–σ orbital of bromoacetate anion, n(H_2_O)–non-bonding orbital of water, n(Br)–non-bonding orbital of bromide anion, π(phen)–π orbital of phenanthroline, σ(phen)–σ orbital of phenanthroline, *–an antibonding orbital.

**Table 3:** Crystal data and structure refinement details for the studied compounds.

Compound	1	2
Empirical formula	C_16_H_14_Br_2_CuN_2_O_5_	C_32_H_25_Br_5_Cu_2_N_4_O_8_
Formula weight	537.65	1120.19
Crystal system	Triclinic	Monoclinic
Space group	*P*-1 (No. 2)	*C*2/*c* (No. 15)
Unit cell dimensions		
a (Å)	9.1666(2)	25.3799(7)
b (Å)	10.4887(2)	7.2326(2)
c (Å)	11.0716(3)	20.1769(5)
α (°)	65.382(2)	90.00
β (°)	66.608(2)	107.993(3)
γ (°)	73.039(2)	90.00
Volume (Å^3^)	877.95(4)	3522.59(17)
Z	2	4
Calculated density (Mg/m^3^)	2.034	2.112
Absorption coefficient (mm^−1^)	7.371	6.918
*F(000)*	526	2168
Crystal size (mm)	0.060 × 0.095 × 0.179	0.016 × 0.038 × 0.242
*θ* Range for data collection (°)	4.632 to 78.742	2.940 to 31.436
Index ranges	−11 ≤ h ≤ 11, −13 ≤ k ≤ 12, −13 ≤ l ≤ 13	−36 ≤ h ≤ 34, −10 ≤ k ≤ 10, −28 ≤ l ≤ 26
Reflections collected / unique	40793/3603	30256/5249
R*_int_*	0.0307	0.0471
Completeness (%)	99.9 (to *θ* = 67°)	99.9 (to *θ* = 25°)
Min. and max. transmission	0.500 and 0.973	0.404 and 1.000
Data / restraints / parameters	3603/0/241	5249/0/234
Goodness-of-fit on *F*^2^	1.083	1.013
Final *R* indices [*I* > 2σ(*I*)]	*R*1 = 0.0211, *wR*2 = 0.0535	*R*1 = 0.0320, *wR*2 = 0.0756
R indices (all data)	*R*1 = 0.0215, *wR*2 = 0.0537	*R*1 = 0.0448, *wR*2 = 0.0802
Largest diff. peak and hole (e•Å^−3^)	0.846 and −0.917	1.869 and −1.368

## Supplementary Materials

The following are available online. Supplemental experimental section, Supplemental discussion, Table S1: Hydrogen bonds in the studied compounds. Table S2: Stacking interactions in the studied compounds. Table S3: The energy of 2 calculated for its various spin states. Table S4: Vibrational frequencies and their assignments for the studied compounds. Figures S1–S5: Hirshfeld surfaces and 2D decomposition fingerprints of the studied compounds. Figure S6: Stacking interactions in the studied compounds. Figures S7–S8: Hirshfeld surfaces and 2D decomposition fingerprints of literature known compound (CSD refcode: NOQPEP). Figure S9: Three-dimensional fluorescence spectra of the studied compounds. Figure S10: ATR-IR spectra of the studied compounds. Figure S11: TG, DTA, and DTG curves for the studied compounds. Figure S12–S13: Mass spectra of volatile products from the thermal decomposition of the studied compounds. Figure S14: Experimental and calculated UV-Vis spectra of 2.
