# Supplementary material for: Physicochemical Insight into Coordination Systems Obtained from Copper(II) Bromoacetate and 1,10-Phenanthroline
Source: Molecules. 2020 Nov 15;25(22):5324. doi: 10.3390/molecules25225324 (PMC7697942; doi:10.3390/molecules25225324)
Supplement: Supplementary file 1 [file molecules-25-05324-s001.pdf]

Supplementary Materials

Physicochemical insight into coordination systems obtained from copper(II) bromoacetate and 1,10-phenanthroline

Ewelina Krejner, Tomasz Sieranski, Marcin Swiatkowski, Marta Bogdan and Rafal Kruszynski

Supplemental experimental section

Supplemental synthesis description

The synthesis of $[Cu(BrCH_2COO)_2(phen)]$ compound in hydrothermal conditions is described in the literature (main manuscript reference no. [25]). Authors of the original paper provide the following synthesis description: "*The reaction was carried out by the solvothermal method. 2-bromoacetic acid (0.104 g, 2 mmol) and cupric acetate (0.199 g, 1 mmol) and 1,10-phenanthroline (0.180 g, 1 mmol) were added to the airtight vessel with 20 ml water. The resulting green solution was filtered. The filtrate was placed for several days yielding blue block-shaped crystals.*" [25].

Because only the amounts of substances and solvent were given, the synthesis was repeated at different conditions. The substrates and solvent in above mentioned amounts were placed in the stainless steel reactors with teflon interior and following volumes 25 cm^3, 50 cm^3, and 100 cm^3. The reactors were placed for 24 hours in an oil bath with temperature set to 433 K. After process the all reactors continued clear solutions, which were transferred to beakers and left for crystallisation. After a 1-3 days in all beakers the $[CuBr(phen)_2]^+$•Br^- hydrate [23] and Cu(oxalate) were formed. Because used conditions led to total decomposition of bromoacetate ions the experiments were repeated at more mild setup, i.e. the all variations of following temperature (temp.) and time sets were used: temp. = {373, 393, 413 K}, time = {2, 4, 6, 12 h}. Nevertheless of used conditions, the mixture of the $[CuBr(phen)_2]^+$•Br^- hydrate and Cu(oxalate) was created in all cases.

Methodology of the bond valence calculation

The bond valences were calculated as $v_{ij} = \exp[(R_{ij}-d_{ij})/b]$ [30, 31]. The R_{ij} is the bond-valence parameter that formally reflects the idealized single-bond length between *i* and *j* atoms for the given *b*. The *b* was taken as 0.37 Å [32]. For coordination number (c.n.) = 5 $R_{Cu\text{-}N}$, $R_{Cu\text{-}O}$, $R_{Cu\text{-}Br}$ were 1.705, 1.652, 2.136 Å, respectively, and for c.n. = 6 $R_{Cu\text{-}N}$, $R_{Cu\text{-}O}$ were 1.715, 1.656 Å, respectively [33]. The computed bond valences are collected in Table 2.

Supplemental discussion

Analysis of Hirshfleld surfaces and fingerprints

The fingerprint plots of the outer coordination sphere of **2** (Figures S3 and S5) are strongly asymmetric (there is no mirror symmetry along the $d_e = d_i$ diagonal) due to the presence of different species around the supramolecular dimer (complex moieties and other dimers). Such variety cause that the distances from the surface point to the nearest nucleus in another molecule and in the molecule itself are different. This effect is relatively common for compounds with Z'> 1 as in such cases, not all molecules are related by crystal symmetry, and consequently, not all d_e, d_i pairs have equivalent d_e', d_i' pairs in which $d_e' = d_i$ and $d_i' = d_e$. The relatively high symmetricity of the fingerprints of **1** and the coordination unit of **2** (Figures S1, S2, and S4) originates from the presence of only one molecule in the asymmetric unit of **1** and the presence of internal symmetry in the complex molecule of **2** (what subsequently cause similarity of surrounding of this molecule). The H•••O contacts significantly contribute to the Hirshfeld surfaces of the studied compounds (Figures S1-S5). This contribution is larger for coordination units of

1 at about 7% (Figures S2, S4). The Hirshfeld surfaces also indicate the significance of C-H•••Br interactions. The contribution of H•••Br contacts is comparable to the H•••O contacts (Figures S2, S4, and S5), and the exact percentage is even higher for the coordination unit of **2** as a result of the presence of three bromine atoms in this moiety (Figure S4). The H•••Br contacts involve phen ligands, bromoacetate ions, and in the case of **2**, the neutral bromoacetic acid molecule and bromide ion.

The *π*•••*π* interactions are represented as the flat region of the surface above phen rings in curvedness plots (Figures S1 and S3) and as orange-red areas above phen rings in shape index plots (Figures S1 and S3). These rings interactions are principally expressed in Hirshfeld analysis as C•••C interactions and secondary as C•••H and N•••H interactions between phen rings. The contribution of C•••C interactions into the total surface is about 2% greater in **2** than in **1**. It is in agreement with the above described total number of π•••π interactions formed by six-membered rings of phen (14 and 12 respectively for **2** and **1**). The H•••N contacts are of lesser significance, and they participate in 2-3% of the total surfaces. A large contribution of H•••H contacts to the total surface (Figures S2, S4, and S5) is observed in the studied compounds due to the hydrogen atoms' approach.

The Hirshfeld surfaces (as well as its 2D decomposed fingerprints, Figures S7, S8) of the known bis(2-bromoacetato-κ^2*O,O′*)(1,10-phenanthroline-κ^2*N,N′*)copper(II) [25] shows partial similarity. However, some obvious differences exist. The 2D fingerprint map of bis(2-bromoacetato-κ^2*O,O′*)(1,10-phenanthroline-κ^2*N,N′*)copper(II) is more condensed than of **1**, i.e. the d_e and d_i distances ranges are about 0.5 Å smaller. The order of dominant contacts is also different (the H•••H precedes O•••H in **1** while in the known compound [25] H•••H succeeds O•••H) and the all percentage contributions varies (e.g. H•••Br in **1** differ about 4% from the value calculated for the known compound [25]; Figures S2 and S8).

Table S1. Hydrogen bonds in the studied compounds (Å, °).

D-H•••A	d(D—H)	d(H•••A)	d(D•••A)	<(DHA)
compound **1**				
O5—H5O•••O2	0.80(3)	1.88(3)	2.643(2)	159(3)
O5—H5P•••O3^{i}	0.74(3)	2.02(3)	2.760(2)	171(3)
C1—H1•••O1	0.95	2.49	2.986(2)	112.7
C2—H2•••Br2^{ii}	0.95	3.05	3.820(2)	138.8
C11—H11•••Br1^{i}	0.95	3.13	4.082(2)	175.9
C12—H12•••O2^{i}	0.95	2.59	3.302(3)	131.9
C14—H14A•••O4^{ii}	0.99	2.42	3.091(3)	124.8
C14—H14B•••O1^{ii}	0.99	2.61	3.454(3)	142.8
C16—H16A•••O4^{i}	0.99	2.48	3.458(2)	170.5
compound **2**				
O22—H22O•••O22^{iii}	0.91(9)	1.58(8)	2.457(4)	160(9)
C1—H1•••Br21^{iv}	0.95	3.10	3.821(3)	134.2
C2—H2•••Br1^{v}	0.95	3.12	4.006(3)	156.2
C11—H11•••O21	0.95	2.45	3.056(3)	121.9
C12—H12•••O1	0.95	2.49	2.963(3)	111.1
C11—H11•••O21	0.95	2.47	3.074(3)	121.6
C14—H14B•••O22^{iii}	0.99	2.55	3.321(3)	135.1
C22—H22A•••O21^{iii}	0.99	2.48	3.301(4)	140.1

Symmetry transformations used to generate equivalent atoms: (i) -x+2, -y+1, -z+1; (ii) x+1, -y+1, -z+2; (iii) -x+0.5, -y+0.5, -z+1; (iv) x+0.5, -y+1.5, z+0.5; (v) x+0.5, y+0.5, z.

Table S2. Stacking interactions in the studied compounds. Each ring is indicated by one atom, which belongs solely to this ring. The α is a dihedral angle between planes I and J, β is an angle between Cg(I)-Cg(J) vector and normal to plane I, d_p is a perpendicular distance of Cg(I) on ring J plane.

R(I)•••R(J)	d(Cg•••Cg) (Å)	α (°)	β (°)	d_p (Å)
compound **1**				
N1•••N1^{i}	3.5481(11)	0.00(10)	20.0	3.3344(8)
N1•••C6^{i}	3.5461(13)	2.29(10)	21.5	3.3441(8)
C6•••N1^{i}	3.5461(13)	2.29(10)	19.4	3.2985(9)
N1•••N2^{i}	4.4545(12)	2.65(10)	41.7	3.3543(8)
N2•••N1^{i}	4.4545(12)	2.65(10)	41.1	3.3265(8)
C6•••C6^{i}	4.9186(14)	0.02(10)	46.5	3.3844(9)
N2•••N2^{ii}	3.6294(12)	0.00(10)	23.8	3.3195(8)
N2•••N1^{ii}	4.8584(12)	2.65(10)	46.0	3.2680(8)
N1•••N2^{ii}	4.8584(12)	2.65(10)	47.7	3.3739(8)
N2•••C6^{ii}	3.5426(13)	0.37(10)	20.2	3.3229(8)
C6•••N2^{ii}	3.5426(13)	0.37(10)	20.3	3.3237(9)
C6•••C6^{ii}	4.8599(14)	0.02	46.7	3.3335(9)
compound **2**				
N1•••N2^{iii}	4.1618(15)	3.95(13)	33.4	3.3513(11)
N2•••N1^{iii}	4.1619(15)	3.95(13)	36.4	3.4762(10)
N1•••C6^{iii}	5.7672(16)	1.51(12)	51.5	3.5017(11)
C6•••N1^{iii}	5.7673(16)	1.51(12)	52.6	3.5908(11)
C6•••N2^{iii}	3.6852(15)	2.54(12)	20.8	3.4022(11)
N2•••C6^{iii}	3.6852(15)	2.54(12)	22.6	3.4453(10)
C6•••C6^{iii}	4.9174(15)	0.02(12)	44.6	3.5030(11)
N2•••N2^{iii}	3.8296(15)	0.00(12)	25.7	3.4518(10)
N1•••N2^{i}	4.5078(15)	3.95(13)	40.9	3.3990(11)
N2•••N1^{i}	4.5079(15)	3.95(13)	41.1	3.4082(10)
N1•••C6^{i}	5.2606(16)	1.51(12)	51.2	3.3264(11)
C6•••N1^{i}	5.2607(16)	1.51(12)	50.8	3.2935(11)
C6•••N2^{i}	3.5502(15)	2.54(12)	17.7	3.3481(11)
N2•••C6^{i}	3.5503(15)	2.54(12)	19.4	3.3828(10)
C6•••C6^{i}	3.8233(16)	0.02(12)	29.6	3.3251(11)
N2•••N2^{i}	4.7198(15)	0.00(12)	45.7	3.2985(10)

Symmetry transformations used to generate equivalent atoms: (i) -x+1, -y+2, -z+1; (ii) -x+2, -y+2, -z+1; (iii) -x+1, -y+1, -z+1.

Table S3. The energy of **2** calculated for its various spin states.

State	Energy (hartee)
singlet	-18195.75777870
triplet	-18195.80215160
open shell singlet	-18195.80223680
antiferromagnetic singlet	-18195.82096720

Table S4. Vibrational frequencies (cm^{-1}) and their assignments for the studied compounds

1	2	Na($BrCH_2COO$) [39]	phen [40, 41]	Assignment
3251 br				ν OH (H_2O)
3084 w	3089 w		3058 w	ν CH
3054 w	3062 w		3035 w	ν CH
3028 w	3019 w	3007 m		ν_{as} CH_2
2968 w	2955 w	2969 m		ν_s CH_2
	1738 m	1720 s [49]		ν C=O (COOH)
1625 s			1638 s	ν CC, ν CN
1608 s	1615 s	1590 s		ν_{as} COO
1590 w	1590w		1597 w	ν CC, ν CN
1583 w			1585 w	ν CC, ν CN
1518 m	1517 m		1504 s	ρ CH
1492 w	1493 w		1492 w	ρ CH
1451 w	1457 w			ρ CH
	1427 m		1420 s	ν CC, ν CN, δ-α CH
1426 s	1411 s	1408 s		ν_s COO
1411 w				ν CC, ν CN, δ-α CH
1398 m	1396 s	1383 s		σ CH_2
1373 s				ν CC, ν CN, δ-α CH
1352 s				ν CC, ν CN, δ-α CH
1340 s	1340 w		1345 m	ν CC, ν CN, δ-α CH
	1317 w			ν CC, ν CN, δ-α CH
1254 w	1253 w		1294 w	ν CC, ν CN, δ-α CH
	1224 m	1220 s	1218 m	ω CH_2, ω CH
1209 s	1208 w	1209s		ω CH_2
1146 w			1142 m	δ-γ CH
1118 w				δ-γ CH
1106 w	1107 w			δ-γ CH
1092 w	1094 w		1092 m	δ-γ CH
1051 w	1053 w			δ-γ CH, ar breathing mode
1034 w	1036 w		1035 w	δ-γ CH, ar breathing mode
1007 w	1010 w			δ-γ CH, ar breathing mode
992 w	994 w		996 w	ar breathing mode
969 w	980 w		987 w	ar breathing mode
931 w	954 w		960 w	ar breathing mode
909 w	912 w	926 w		ν CC
895 w		899 s		ρ CH_2
874 w	874 m			ρ CH_2
848 s	854 s		855 s	ω CH
808 w	830 w			ω CH
775 w			786 w	τ CH
	741w			τ CH
738 w	731 w			τ CH
721 s	720s		738 s	τ CH
	695 w		692 w	σ ar
686 s	680 m	696 s		ν CBr
676 s				ν CBr
648 s	641 m	667 m		σ COO
601 w			622 m	ρ ar
	568 w	566 w		ω COO
554 m	549 w		552 m	δ-γ ar
504 w	508 w		508 w	δ-γ ar
491 w	493 w		495 w	δ-γ ar
480 w				δ-γ ar
445 w				ν M—L
429 m	432 m			ν M—L
413w				ν M—L

Vibrations symbols: w – weak, m – medium, s – strong, br – broadened, ν – stretching, δ – bending, ρ – rocking, σ – scissoring, τ – twisting, ω – wagging, s – symmetric, as – asymmetric, α – in-plane, γ – out-of-plane, ar – aromatic ring, M – metal, L – ligand.

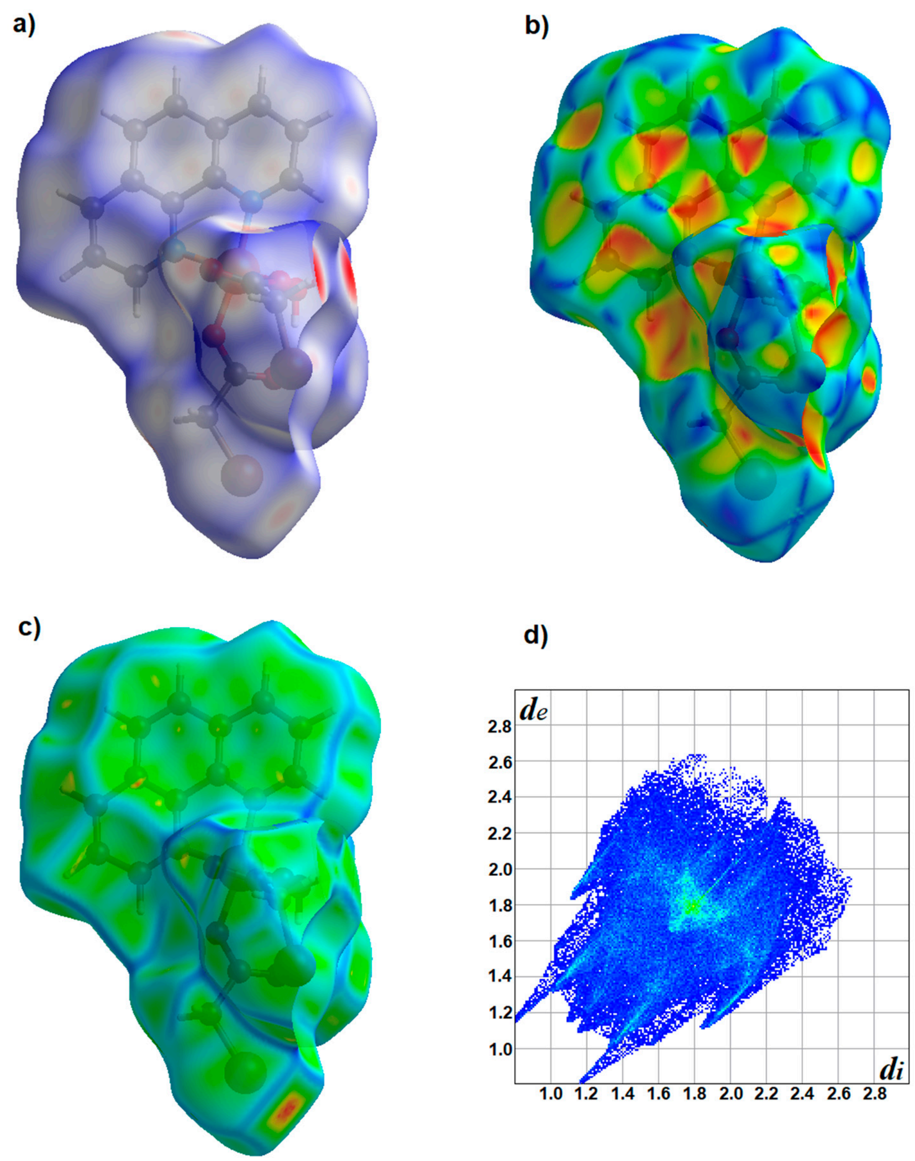

Figure S1. Hirshfeld surfaces of **1** plotted over d_{norm} (a), shape index (b), curvednes (c), and 2D fingerprint map (d).

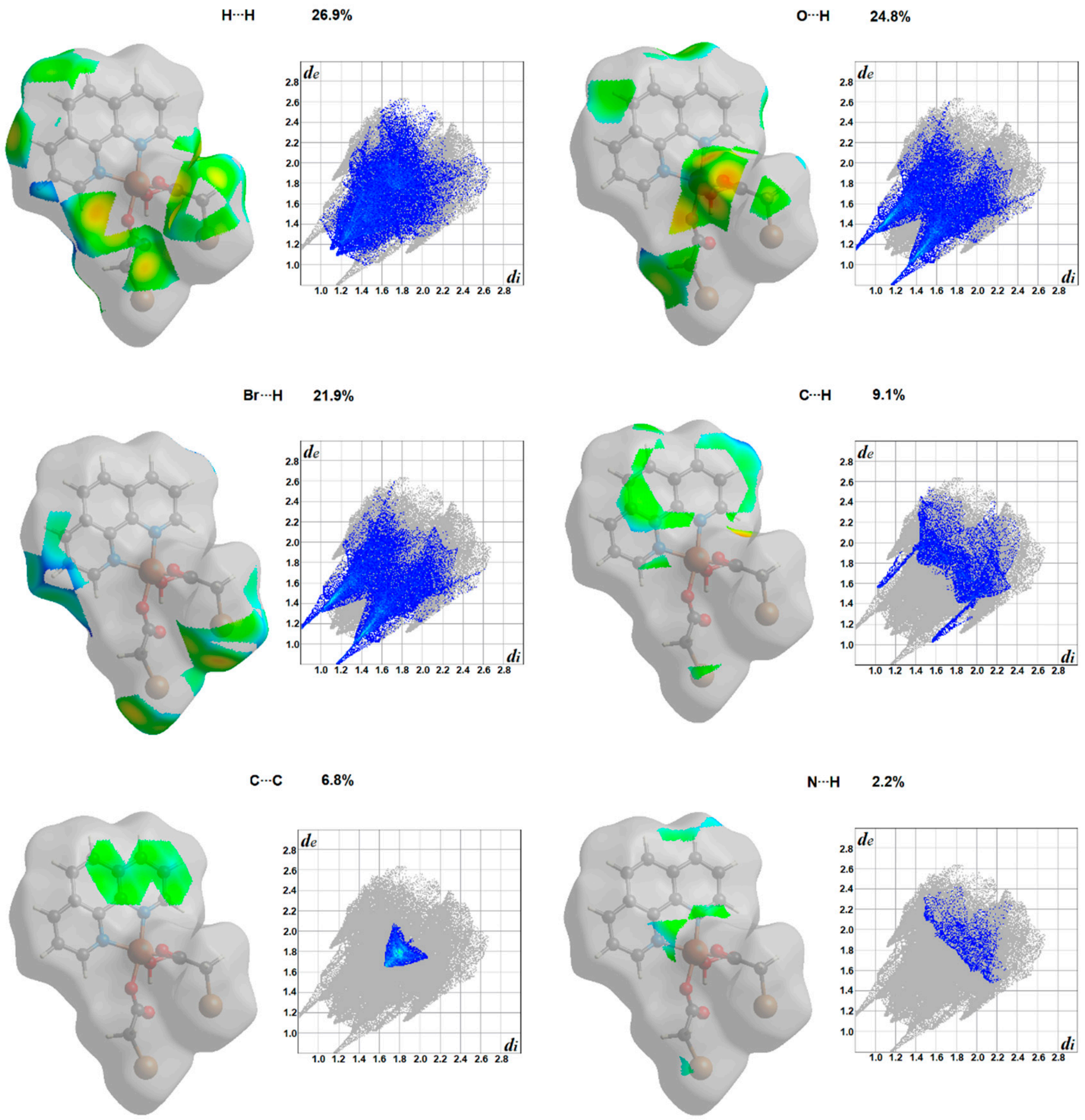

Figure S2. 2D decomposed fingerprints of **1** showing the contributions of atoms within specific interacting pairs. For each fingerprint map, the grey area represents the whole plot. Surface maps (plotted over d_e) next to each fingerprint indicate the areas associated with the specific contact.

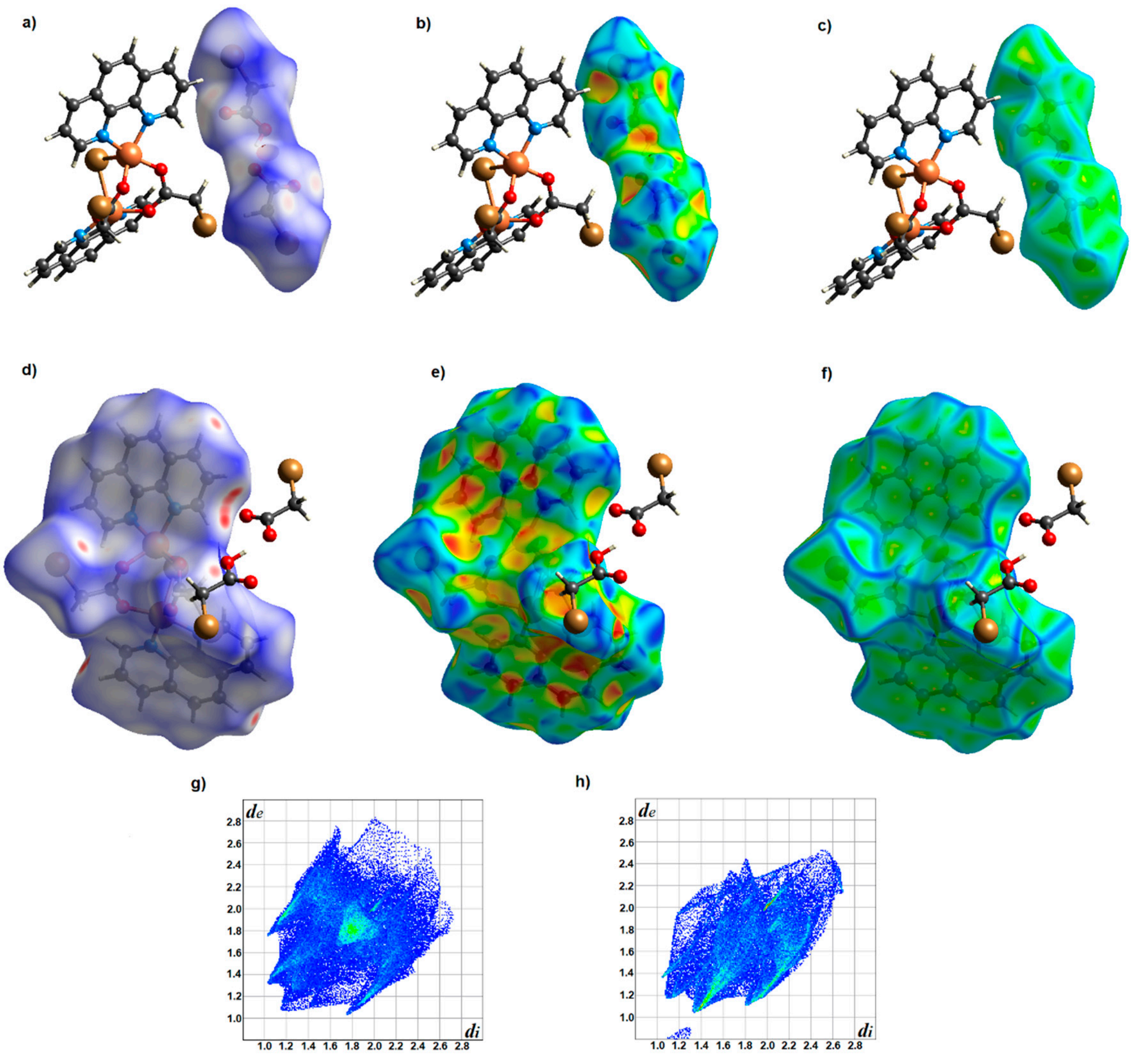

Figure S3. Hirshfeld surfaces of **2** for its interacting bromoacetic acid – bromoacetic ion pair (a-c) and for its coordination unit (d-e). The surfaces were ploted over d_{norm} (a,d), shape index (b, e) and curvedness (c, f). 2D fingerprint maps of the coordination unit **2** (g) and the interacting bromoacetic acid – bromoacetic ion pair (h).

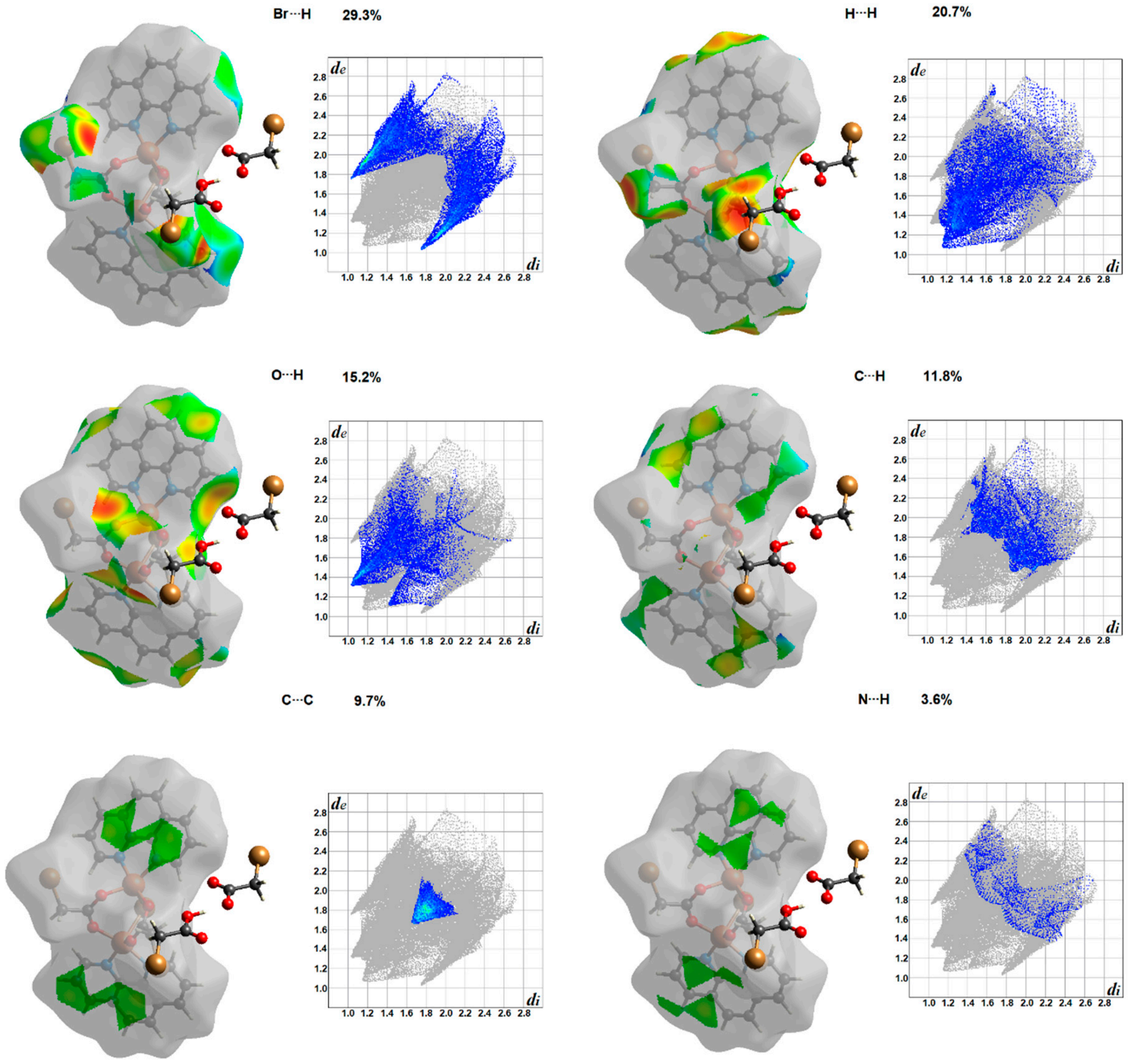

Figure S4. 2D decomposed fingerprints of the coordination unit of 2 showing the contributions of atoms within specific interacting pairs. For each fingerprint map, the grey area represents the whole plot. Surface maps (plotted over d_e) next to each fingerprint indicate the areas associated with the specific contact.

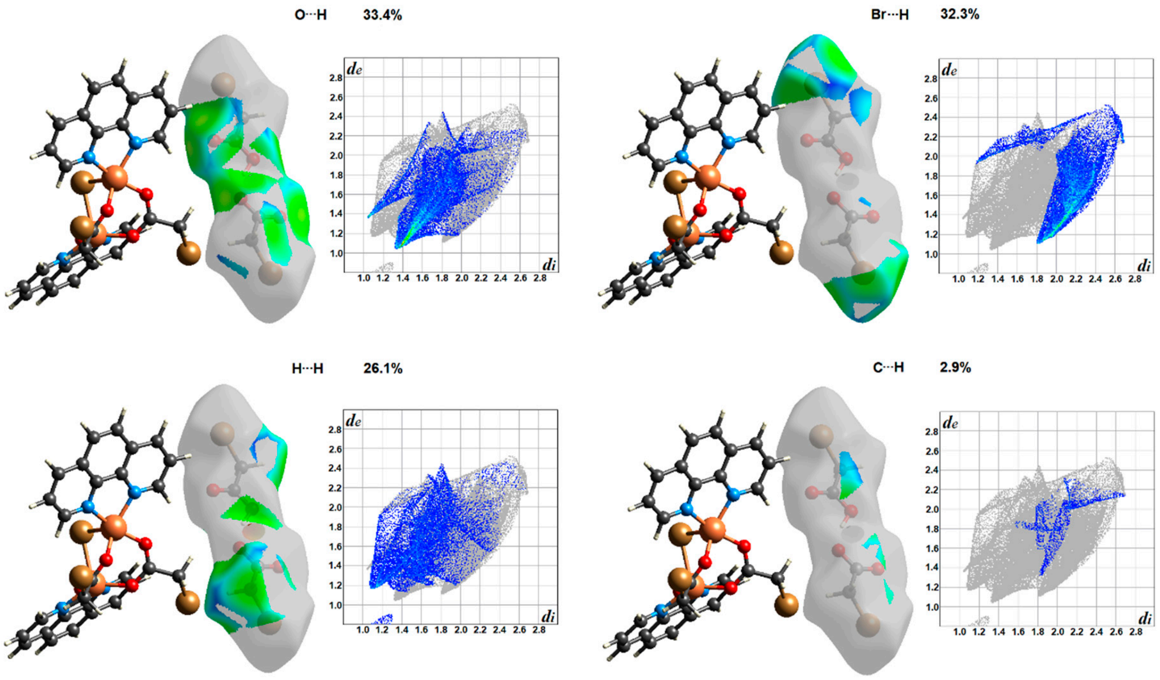

Figure S5. 2D decomposed fingerprints of the interacting bromoacetic acid – bromoacetic ion pair in **2** showing the contributions of atoms within specific interacting pairs. For each fingerprint map, the grey area represents of the whole plot. Surface maps (plotted over d_e) next to each fingerprint indicate the areas associated with the specific contact.

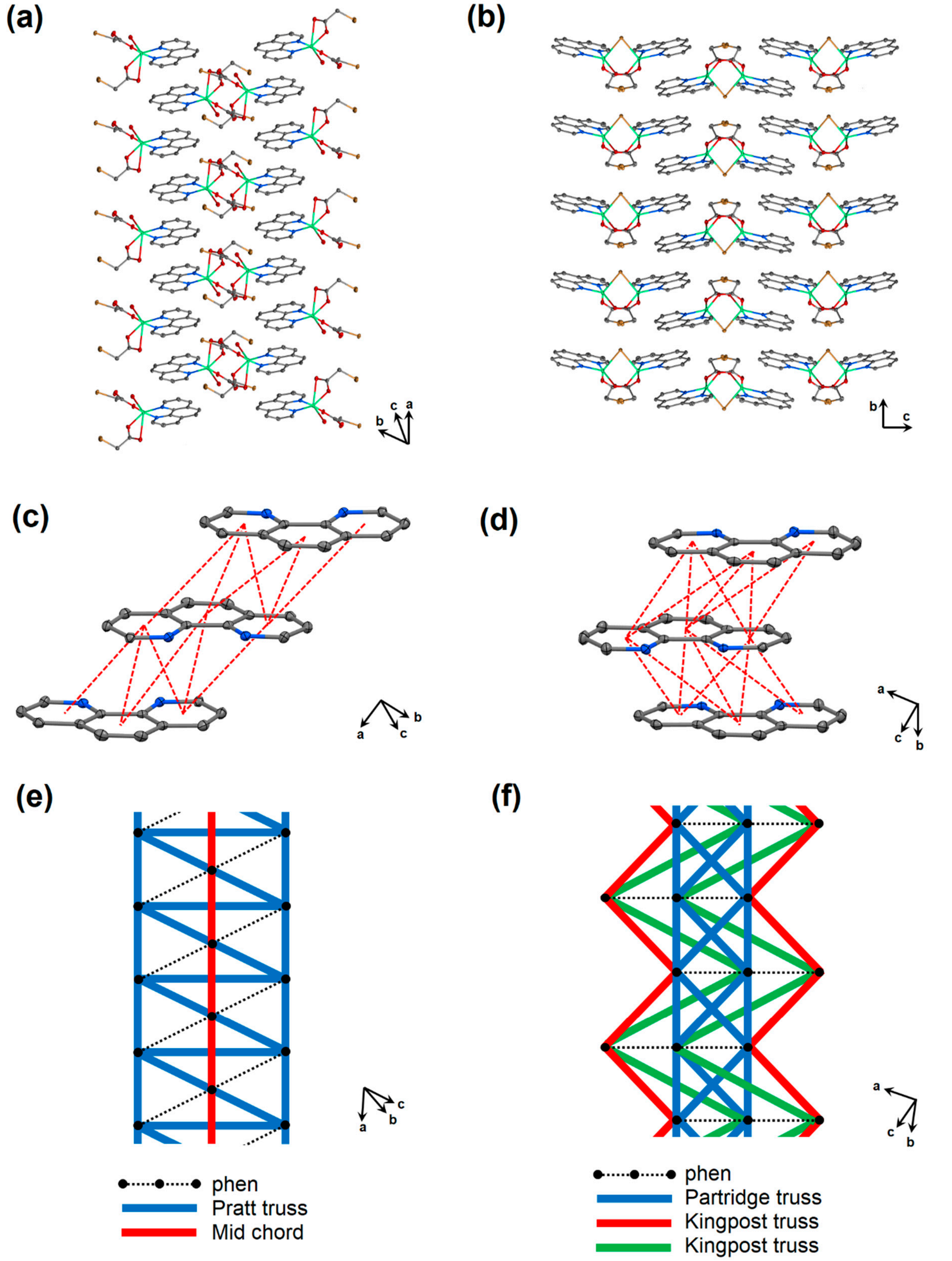

Figure S6. Parts of molecular packing [(a) for compound **1**, (b) for compound **2**], representing the π•••π interactions (dashed lines) between the phen ligands [(c) for compound **1**, (d) for compound **2**], and the simplified interactions schemes [(e) for compound **1**, (f) for compound **2**].

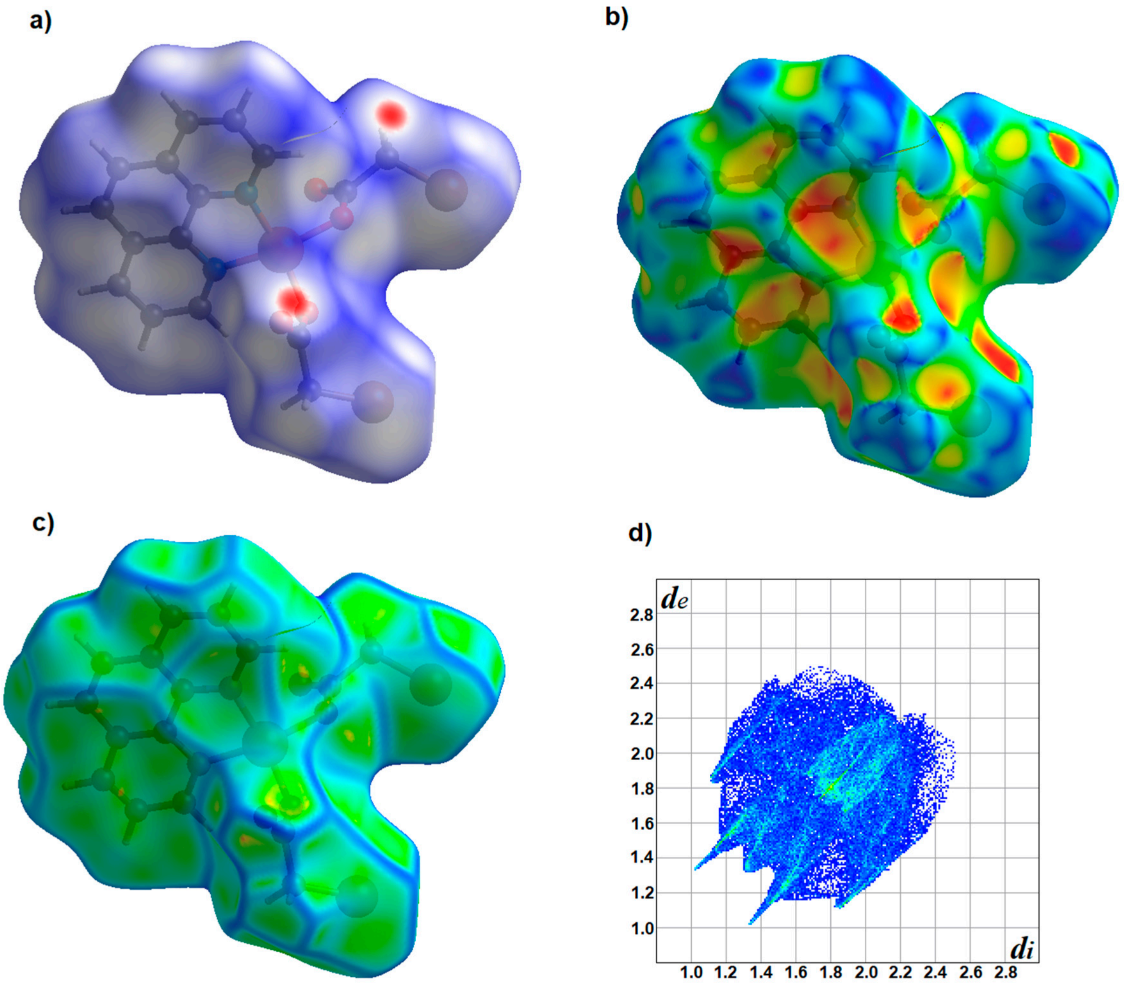

Figure S7. Hirshfeld surfaces of literature known bis(2-bromoacetato-κ^2*O,O′*)(1,10-phenanthroline-κ^2*N,N′*)copper(II) (CSD refcode: NOQPEP) [34] ploted over d_{norm} (a), shape index (b) curvednes (c), and 2D fingerprint map (d).

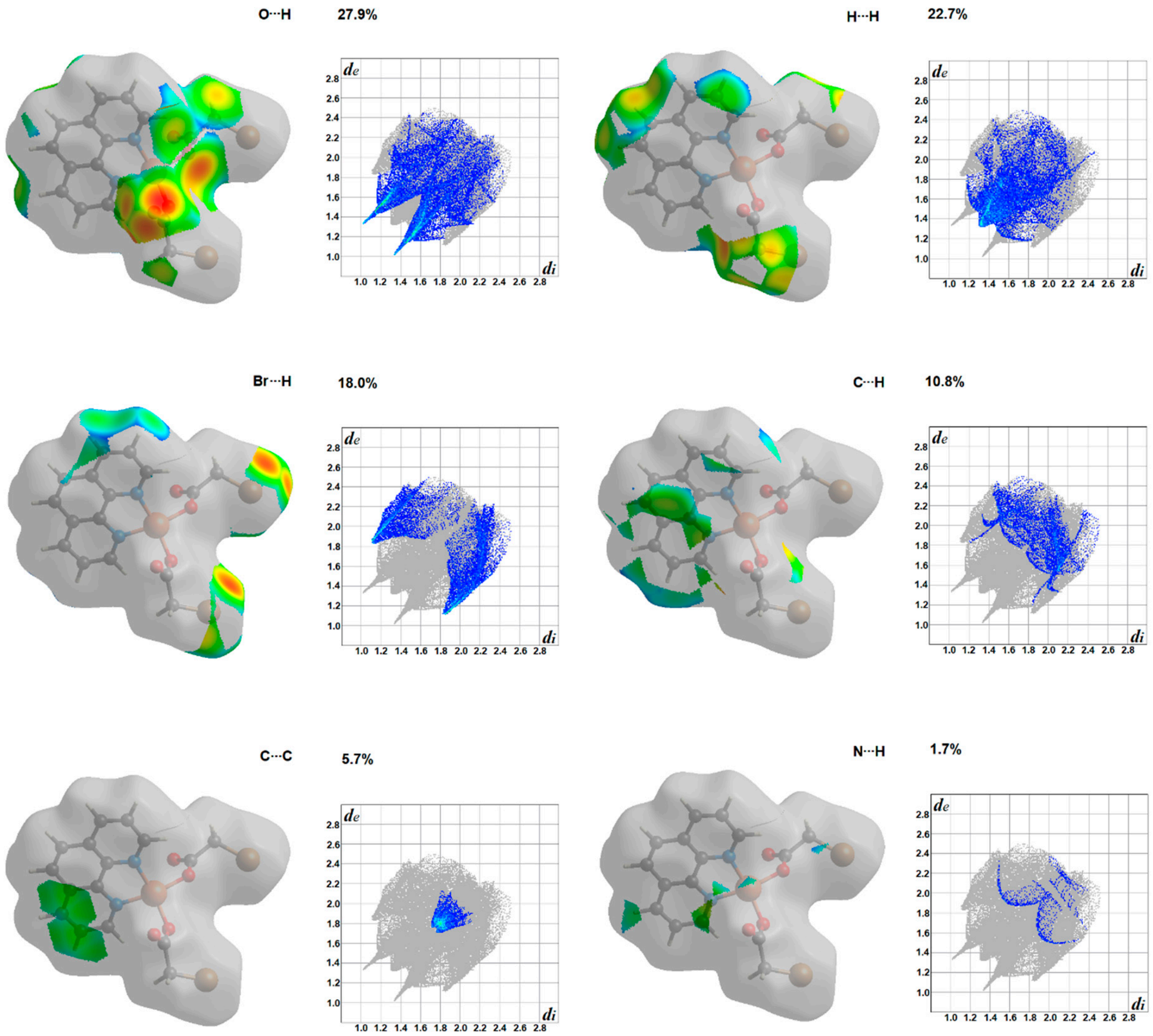

Figure S8. 2D decomposed fingerprints of literature known bis(2-bromoacetato-κ²*O,O′*)(1,10-phenanthroline-κ²*N,N′*)copper(II) (CSD refcode: NOQPEP) [34] showing the contributions of atoms within specific interacting pairs. For each fingerprint map, the grey area is a representation of the whole plot. Surface maps (plotted over d_e) next to each fingerprint indicate the areas associated with the specific contact(s).

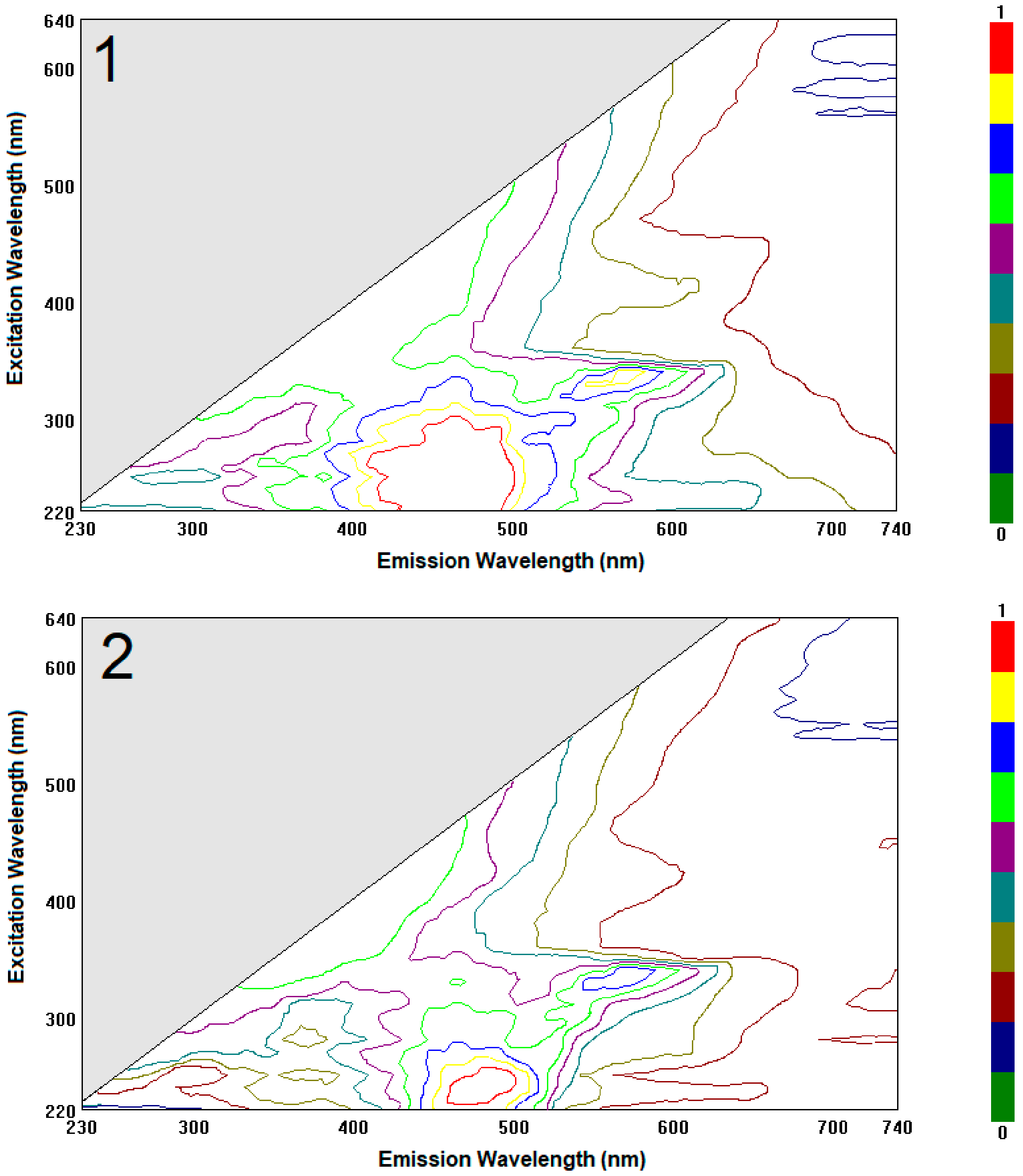

Figure S9. Three-dimensional fluorescence spectra of the studied compounds

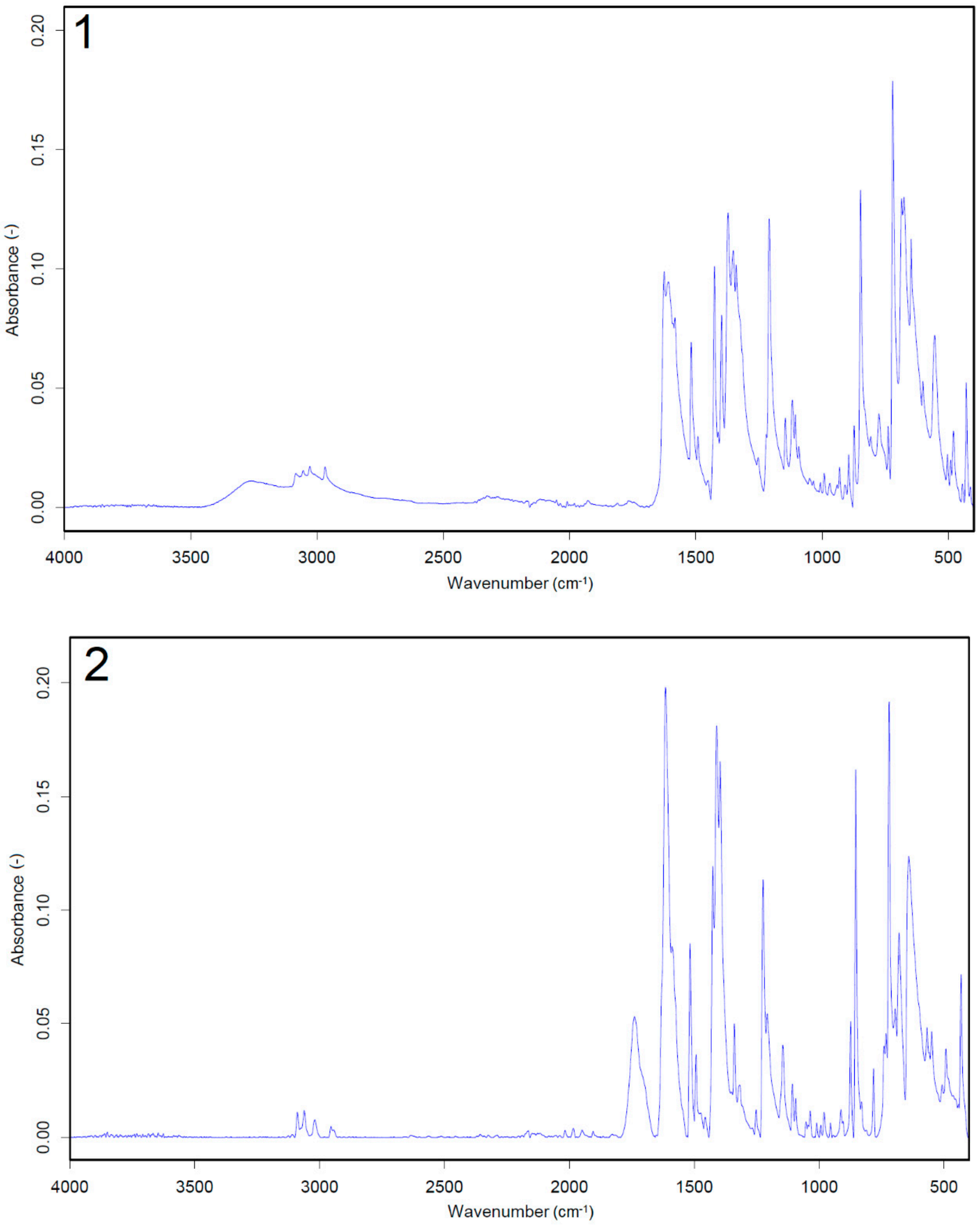

Figure S10. ATR-IR spectra of the studied compounds.

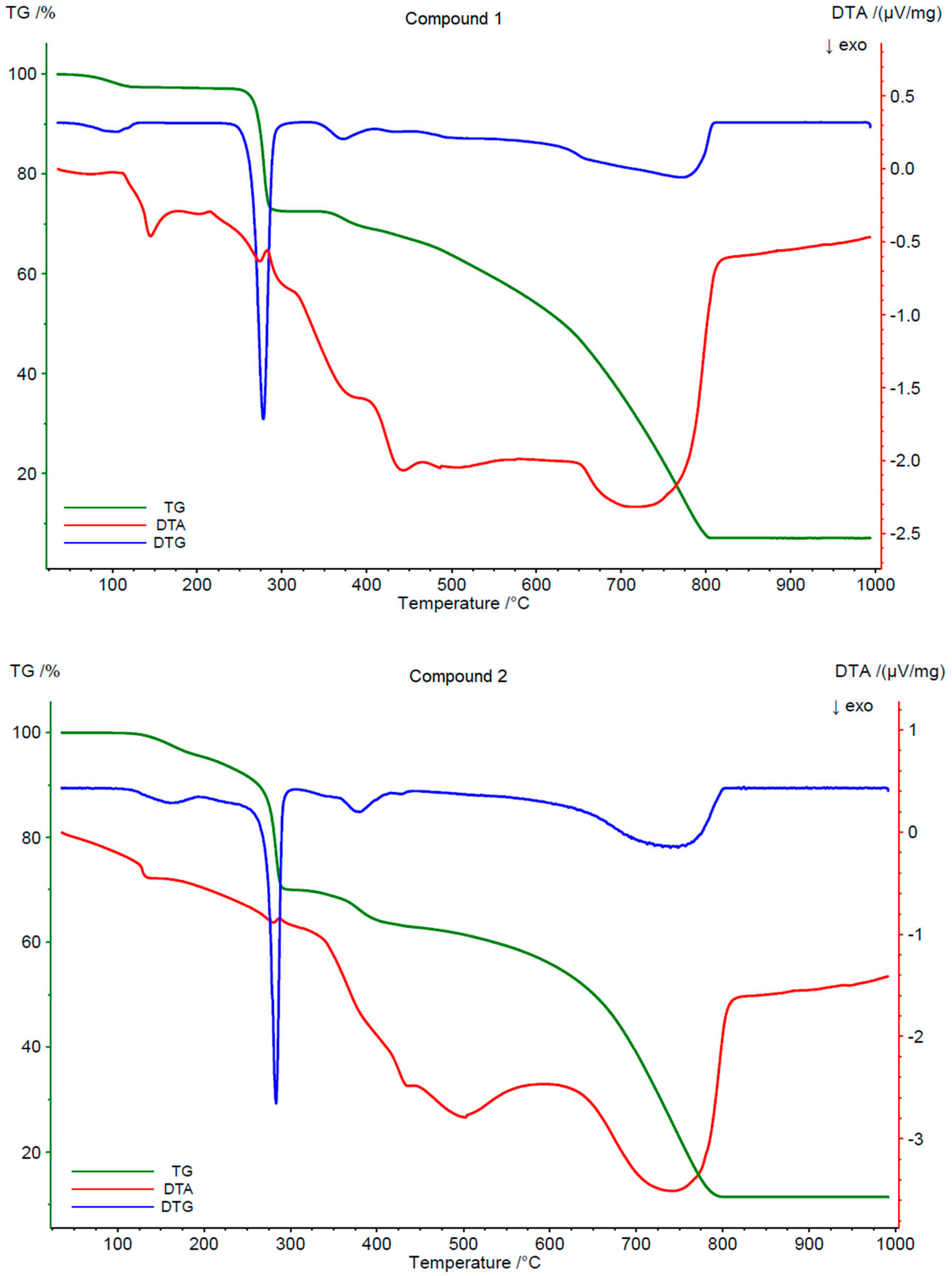

Figure S11. TG, DTA and DTG curves for the studied compounds.

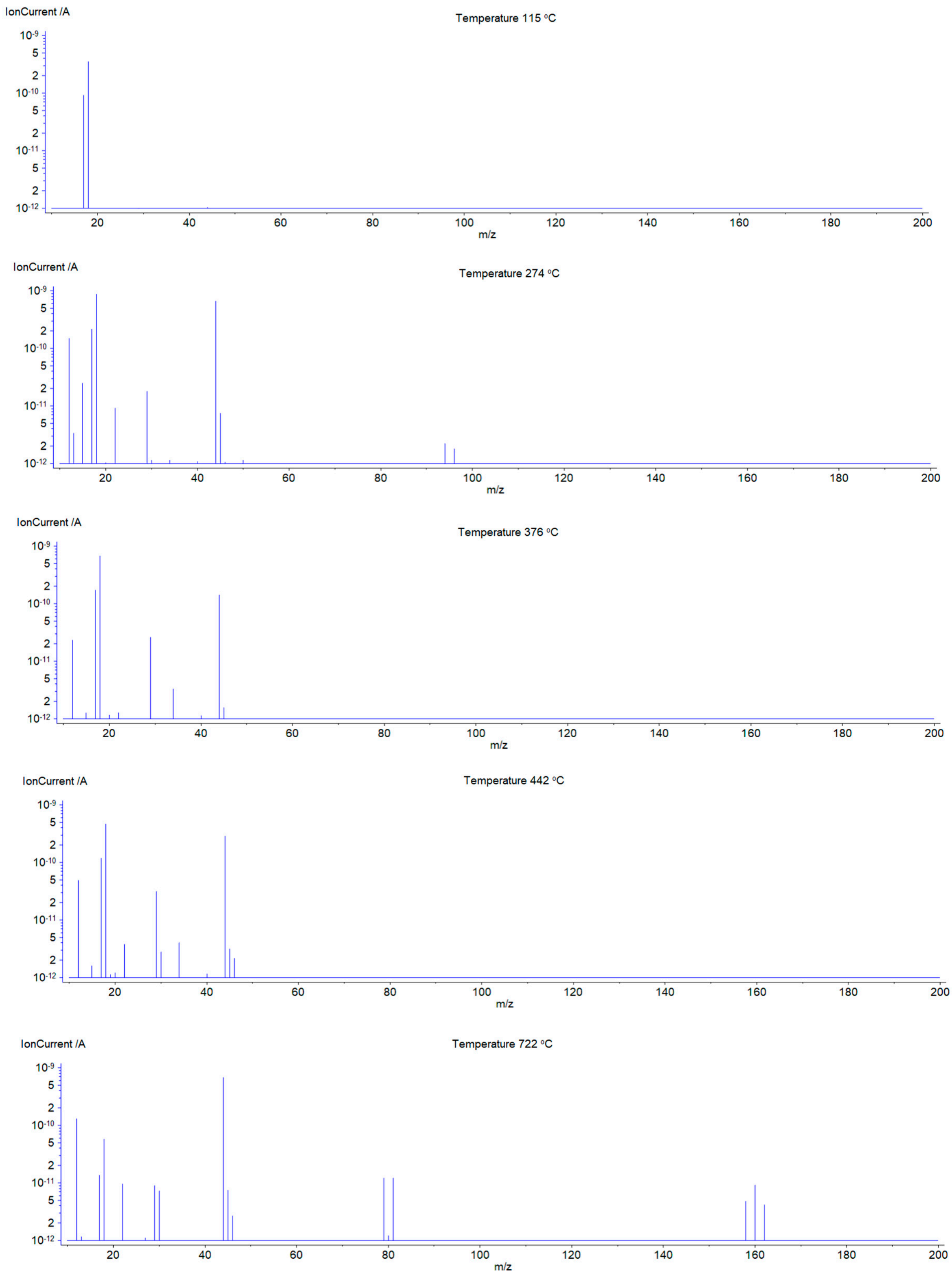

Figure S12. Mass spectra of volatile products from the thermal decomposition of **2**. The elaboration of the mass spectra involved subtracting the background spectrum and application of an automatic software correction for the carrier gas.

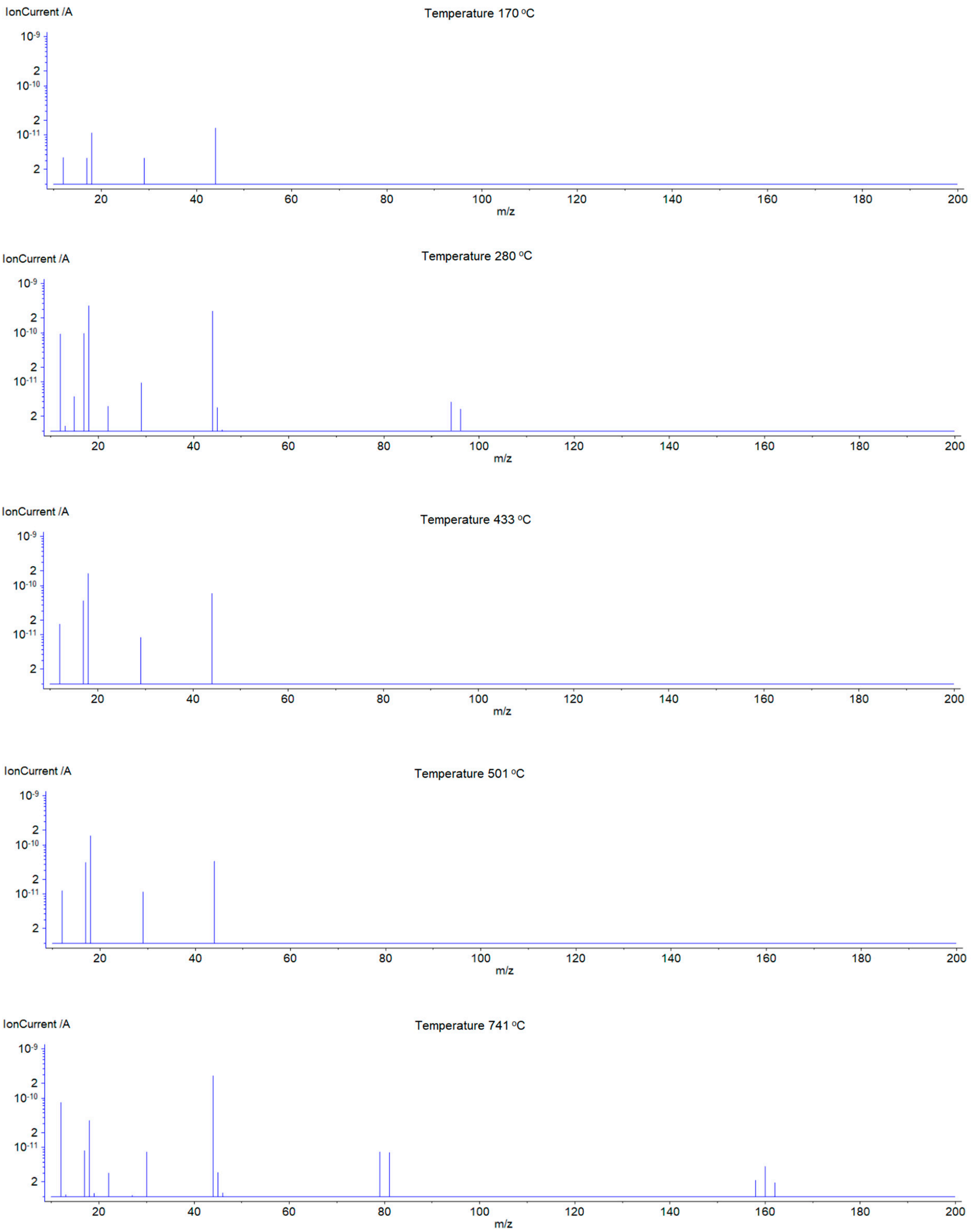

Figure S13. Mass spectra of volatile products from the thermal decomposition of **2**. The elaboration of the mass spectra involved subtracting the background spectrum and application of an automatic software correction for the carrier gas.

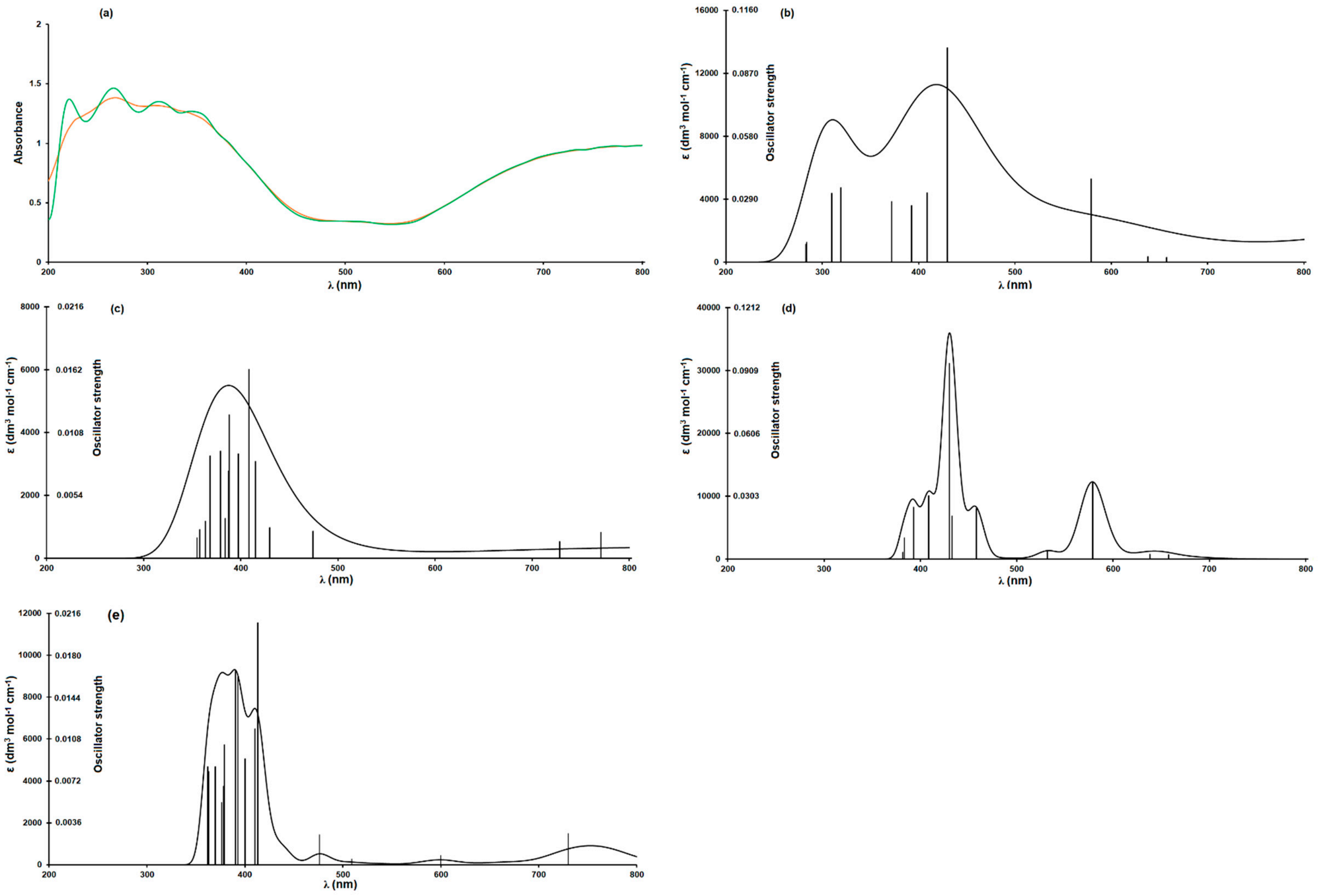

Figure S14. Experimental (a) (orange - registered, green - deconvoluted) and calculated UV-Vis spectra of **2** (b – singlet state, c – triplet state, d – open shell singlet state, e – antiferromagnetic singlet). The most important oscillator strengths are shown as vertical black lines. All calculations were performed at B3LYP/6-31g++(2d, 2p) level of theory.
